# Daytime Napping, Incident Atrial Fibrillation, and Dynamic Transitions With Dementia

**DOI:** 10.1016/j.jacadv.2024.101108

**Published:** 2024-07-12

**Authors:** Chenglong Li, Daijun He, Chao Yang, Luxia Zhang

**Affiliations:** a National Institute of Health Data Science at Peking University, Beijing, China; b Institute of Medical Technology, Health Science Center of Peking University, Beijing, China; c Renal Division, Department of Medicine, Peking University First Hospital, Beijing, China; d Institute of Nephrology, Key Laboratory of Renal Disease, Ministry of Health of China, Beijing, China; e Key Laboratory of Chronic Kidney Disease Prevention and Treatment (Peking University), Ministry of Education, Beijing, China; f Research Units of Diagnosis and Treatment of Immune-Mediated Kidney Diseases, Chinese Academy of Medical Sciences, Beijing, China; g State Key Laboratory of Vascular Homeostasis and Remodeling, Peking University, Beijing, China; h Center for Digital Health and Artificial Intelligence, Peking University First Hospital, Beijing, China

**Keywords:** atrial fibrillation, daytime napping, dementia, genetic predisposition, left ventricular ejection function

## Abstract

**Background:**

Associations between napping and incident atrial fibrillation (AF) remain unknown, and few studies have accounted for dynamic transitions between AF and dementia.

**Objectives:**

The purpose of this study was to evaluate associations between napping with incident AF and the dynamic transitions of AF and dementia, as well as the mediation pathway of left ventricular (LV) size and function.

**Methods:**

A total of 476,588 participants from UK Biobank were included. Napping frequency and other sleep behaviors were evaluated. Incident AF, dementia, and mortality were ascertained via linkage to external registry databases. LV size and function indices were obtained from cardiovascular magnetic resonance imaging phenotypes. A multistate survival analysis was conducted to examine daytime napping in relation to dynamic transitions. Weighed AF genetic risk score was calculated.

**Results:**

Frequent daytime napping, compared to never/rarely napping, was associated with a 1.17-fold AF risk (HR: 1.17; 95% CI: 1.12-1.22), which persisted after controlling for other sleep behaviors. Genetic predisposition significantly modified associations between napping and AF (*P* for interaction <0.001), with stronger associations observed in those of low and moderate genetic risk. LV ejection fraction significantly mediated 26.2% (95% CI: 4.2%-74.1%) of associations between napping and AF. Frequent napping was also associated with a 1.27-fold risk of transition from AF to comorbidity of AF and dementia.

**Conclusions:**

Our findings highlight the potential importance of screening for napping in view of the association with incident AF and dementia. Routine evaluations of the LV ejection fraction could be warranted to timely identify early indications of AF onset among habitual nappers.

Atrial fibrillation (AF) remains the most prevalent type of cardiac rhythm abnormalities. The total number of disability-adjusted life years due to AF has increased from 3.79 million in 1990 to 8.39 million in 2019, while the number of prevalent cases has nearly doubled to 59.7 million since 1990.^1^ Incident AF was associated with elevated risks of cardiovascular events and mortality.^2^ More importantly, as most AF cases, featured by asymptomatic heart rhythm abnormalities, are undetected in practice, the related health impacts can be significantly underestimated,^3^ highlighting the importance of primary prevention.

Dementia accounts for substantial proportions of mortality and disability around the world. Notably, previous studies have associated AF diagnosis with dementia risk, with potential mechanisms included hypoperfusion, inflammation, atherosclerotic vascular disease, microhemorrhage, and brain atrophy.^4^, ^5^, ^6^, ^7^ Nevertheless, little is known regarding modifiable risk factors contributing to dynamic transitions between the 2 diseases.

Among identified risk factors, sleep behaviors have attracted much attention. Previous studies have found associations of excessive daytime sleepiness,^8^ sleep quality,^9^ and overall sleep duration^10^ with incident AF or other types of arrhythmias. Another prospective cohort reported associations between better adherence to a healthy sleep pattern and lower AF incidence.^11^ However, few studies have evacuated associations between daytime napping and AF risk. As a common behavior, daytime napping is usually regarded as a direct consequence of inadequate or poor nighttime sleep^12^ and associated with elevated risks of cardiovascular disease, such as heart failure and stroke.^12^^,^^13^ Recent studies also observed relationships between napping and incident dementia.^14^^,^^15^ So far, prospective studies investigating associations between daytime napping and AF risk are still lacking. And no prospective studies have accounted for the dynamic transitions between AF and dementia. In addition, genetic predisposition has been identified as a major risk factor of AF,^16^ with significant interactions observed in associations between healthy sleep patterns and AF onset.^11^ Finally, left ventricular (LV) size and function have been regarded as important parameters impacting clinical practice regarding AF,^17^ while its role in associations between napping and AF remains largely unknown.

Therefore, the current study is to assess associations between daytime napping and AF incidence, as well as dynamic transitions of AF and dementia. We also aim to evaluate the modifying effect of AF genetic predisposition. The potential mediation pathway linking daytime napping and AF incidence will be examined, with indices of LV size and function included as hypothesized mediators.

## Methods

### Study population

Approved by the North West Multi-centre Research Ethics Committee, the UK Biobank was established with the aim to lay foundations for comprehensively investigating risk factors of major chronic diseases. More than 500,000 men and women aged 40 to 69 years from 22 assessment centers in England, Scotland, and Wales were recruited during 2006 to 2010. Before enrollment, all participants provided informed consent, with further details regarding the design and the survey content described elsewhere.^18^

Among the original 502,369 participants available for inclusion, we excluded 8,374 participants with a historical AF diagnosis at baseline, 1870 participants missing assessment of daytime napping, and 15,537 participants without information on AF genetic risk, leaving 476,588 participants for final analysis regarding AF incidence. We also excluded 200 participants with prevalent dementia at baseline, leaving 476,388 participants for dynamic transitions analysis. In addition, we further excluded 438,461 participants without assessment of LV size and function, leaving 38,127 participants in mediation analysis. The detailed sample selection procedure was presented in Supplemental Figure 1.

### Daytime napping and other sleep behaviors

Self-reported information on sleep behaviors in the UK Biobank was obtained via a touchscreen questionnaire. Daytime napping information was collected by asking participants “Do you have a nap during the day?” and a tip was given to restrict the time frame to last 4 weeks if the participants indicated any difficulties in answering the question. Potential responses included: 1) never/rarely have a nap; 2) sometimes have a nap; 3) usually have a nap; and 4) prefer not to answer. The “prefer not to answer” response was coded as missing and therefore excluded from analysis.

In addition to daytime napping, we also accounted for other sleep behaviors, including early chronotype, total sleep duration, insomnia symptoms, snoring, and excessive daytime sleepiness. Chronotype was investigated by asking the question “Do you consider yourself to be: 1) definitely a ‘morning’ person; 2) more a ‘morning’ than ‘evening’ person; 3) more an ‘evening’ than a ‘morning’ person; and 4) definitely an ‘evening’ person.” Responses including “morning” or “morning than evening” were categorized as early chronotype. Sleep duration was investigated by asking “About how many hours sleep do you get in every 24 hours? (please include naps)” and further categorized into 7 to 8 h/day or not. Insomnia symptoms were evaluated by asking “Do you have trouble falling asleep at night or do you wake up in the middle of the night?”, with potential responses including: 1) never/rarely; 2) sometimes; and 3) usually. Then participants were categorized into whether never/rarely or sometimes encounter insomnia symptoms or not. We accounted for snoring (with self-reported snoring) and excessive daytime sleepiness (“often” or “all of the time”).

### Outcome ascertainment

Information regarding AF and dementia diagnosis was obtained via 3 approaches: 1) the International Statistical Classification of Diseases and Related Health Problems-10th Revision (ICD-10) codes, obtained via linkage to hospital admissions records and death certificate records^19^; 2) the procedure codes (OPCS4), which is a statistical classification for clinical coding of hospital interventions and procedures undertaken by the National Health Service; 3) self-reported diagnosis of clinical conditions (for prevalent AF diagnosis only). Further details regarding the ascertainment procedure and codes were presented in Supplemental Tables 1 and 2. We defined the comorbidity of AF and dementia as diagnoses with AF or dementia at first and further the other.

### Genetic risk of atrial fibrillation

Genetic risk estimation of AF in the UK Biobank was performed by calculating the genetic risk score (GRS) of AF. Based on the most recent meta-analysis of a multiethnic genome-wide association study of AF,^20^ the GRS of AF was calculated as the sum of products between identified single nucleotide polymorphisms (SNPs) and corresponding β coefficients in relation to the AF risk. The SNPs were coded as 0, 1, and 2 according to the number of risk alleles, with 134 independent SNPs included in calculating the GRS, shown in Supplemental Table 3. The β coefficients were derived from the external genome-wide association study.^20^ According to sample tertiles of calculated GRS, participants were categorized as low, moderate, and high genetic risk.^11^

### Left ventricular size and function

Released as a part of the UK Biobank multi-modal imaging study, the indices of LV size and function were obtained from the cardiovascular magnetic resonance (CMR) data.^21^ The detailed protocol for the CMR procedure can be accessed elsewhere.^22^ Due to the efforts of the UK Biobank imaging team, standardized CMR imaging phenotypes were released for further analysis. We considered 6 indices, including the LV ejection fraction, the LV end-diastolic volume, the LV end-systolic volume, the LV stroke volume, the cardiac output, and the cardiac index.

### Covariates

Based on previous studies, we selected covariates for adjustment, including demographics (age, sex, and ethnicity), socioeconomic factors (education attainment, employment status, and family income), behaviors (alcohol consumption, physical activity, and smoking), and prevalent major chronic diseases (cardiovascular conditions other than AF, hypertension, diabetes, chronic kidney disease, and cancer).^11^^,^^23^ We also controlled for mediation usage for hypertension, diabetes, and cholesterol.^16^ Further details regarding covariates definition, assessment, and UK Biobank Data-Field ID were provided in Supplemental Table 4.

### Statistical analysis

The mean ± SD was used for continuous variables, and numbers and percentages for categorical variables. Differences between daytime napping groups were tested using analysis of variance and chi-squared test.

We used the Cox proportional hazards models to estimate the HRs and 95% CIs of incident AF risk. Person-years were calculated from baseline until the date of incident AF or death or loss to follow-up, or December 31, 2022, whichever came first. The weighted Schoenfeld residual was used to examine the underlying proportional hazard assumption, and no significant violations for the variables included were observed (*P* > 0.05).^24^ We included an interaction term of daytime napping and AF genetic risk in the Cox model, to test potential interactions between napping and AF genetic risk on the multiplicative scale. In addition to the relative hazard assessed using Cox models, we also calculated covariates-adjusted AF incidence rate per 1,000 person-years, using the Poisson regression.^25^

To comprehensively evaluate the potential impact of daytime napping on dynamic transitions of AF and dementia, we further conducted a multistate survival analysis using the multistate Markov model. The multistate model is an extension of traditional Cox proportional hazards model, capable of handling multiple competing events as states of transitions and assessing the associations of risk factors with different stages of disease transition simultaneously. The model has been well-embraced for assessing disease transition trajectory in epidemiological studies.^26^ We considered 5 states when building the multistate model, including baseline (free of AF and dementia), incident AF, incident dementia, comorbidity of AF and dementia, and all-cause mortality. Accordingly, 8 transition patterns were predefined: 1) baseline to AF; 2) baseline to dementia; 3) baseline to death; 4) incident AF to comorbidity; 5) incident AF to death; 6) incident dementia to comorbidity; 7) incident dementia to death; and 8) comorbidity to death. For participants with identical recorded date of disease and death, a time-interval of 0.5 day was introduced according to a previous study.^26^ Age was used as the time scale for the analysis, as identical with previous studies.^26^

Mediation pathways were examined using the difference method, with LV size and function indices included as hypothetical intermediate variables. The difference method, applied using the public %MEDIATE SAS macro,^27^ has been broadly embraced for evaluating mediation pathways.^28^ The proportion mediated was calculated by comparing estimates from models with and without the hypothesized intermediate variable.

We also conducted the variable importance analysis to further examine the relative importance of daytime napping for predicting AF risk and incident dementia, along with other sleep behaviors. Calculated using a permutation-based method, the variable importance was defined as the relative change in model predictive performances between data sets with and without permuted values for the associated variable, with higher values indicating higher importance for risk prediction.^29^

Several sensitivity analyses were conducted. First, we further excluded participants with prevalent cardiovascular conditions. Second, we further accounted for other sleep behaviors in analysis, including daytime sleepiness, total sleep duration in hours, chronotype (early chronotype or not), insomnia symptoms (whether usually encounters insomnia symptoms), and snoring. Third, AF cases that occurred within the first 2 years of follow-up were excluded to address reverse causation. Fourth, to account for the competing risk from all-cause mortality, the Fine-Gray model was fitted to reevaluate napping in relation to AF incidence. Fifth, we further controlled for body mass index. Sixth, in addition to the difference method, we further applied the regression-based causal mediation analytical approach,^30^ to examine the robustness of the mediation analysis. Seventh, in addition to AF genetic risk, we also examined interactions between napping and other baseline covariates. Eighth, we further controlled for the 5-item Fried frailty phenotype^31^ and night shift work schedule. Ninth, we excluded individuals developing dementia within 2 years of incident AF in the multistate analysis. Tenth, we further controlled for snoring in trajectory analysis. Finally, to evaluate selection bias, a nonresponse analysis was conducted comparing baseline characteristics of included and excluded participants.

Statistical analysis was conducted using SAS, 9.4 (SAS Institute) and R language 4.3.1 (R Foundation), with a 2-tailed alpha of 0.05 considered statistically significant.

## Results

Shown in Table 1, among the 476,588 participants in UK Biobank (mean [SD] age, 56.5 [8.1] years, 45.3% men), more than half participants never or rarely have a nap (56.5%), 38.3% participants sometimes have a nap, and 5.2% participants usually have a nap. Individuals napping frequently were older, more likely to be men, socioeconomically disadvantaged, less healthy lifestyles, and more prevalent chronic diseases (all *P* < 0.05). The distribution of the calculated AF GRS was presented in Supplemental Figure 2.

**Table 1:** Baseline Characteristics of Participants

	Total Participants (N = 476,588, 100%)	Daytime Napping			*P* Value for Difference^a^
		Never/Rarely (n = 269,038, 56.5%)	Sometimes (n = 182,531, 38.3%)	Usually (n = 25,019, 5.2%)	
Age, y	56.5 ± 8.1	55.4 ± 8.1	57.6 ± 7.9	59.2 ± 7.6	<0.001
Men	216,042 (45.3)	109,571 (40.7)	90,171 (49.4)	16,300 (65.2)	<0.001
White ethnicity	449,501 (94.3)	255,713 (95.0)	170,518 (93.4)	23,270 (93.0)	<0.001
Education attainment					
Lower education	80,489 (16.9)	37,787 (14.0)	36,512 (20.0)	6,190 (24.7)	<0.001
Secondary education	166,579 (35.0)	94,955 (35.3)	63,444 (34.8)	8,180 (32.7)	
Higher education	224,185 (47.0)	133,643 (49.7)	80,219 (43.9)	10,323 (41.3)	
Don’t know or prefer not to answer	5,335 (1.1)	2,653 (1.0)	2,356 (1.3)	326 (1.3)	
Annual household income, £					
<18,000	91,812 (19.3)	43,104 (16.0)	41,293 (22.6)	7,415 (29.6)	<0.001
18,000-30,999	103,462 (21.7)	54,940 (20.4)	42,460 (23.3)	6,062 (24.2)	
31,000-51,999	106,702 (22.4)	63,778 (23.7)	38,182 (20.9)	4,742 (19.0)	
52,000-100,000	83,522 (17.5)	54,803 (20.4)	26,152 (14.3)	2,567 (10.3)	
>100,000	22,167 (4.7)	15,466 (5.7)	6,213 (3.4)	488 (2.0)	
Don’t know or prefer not to answer	68,923 (14.5)	36,947 (13.7)	28,231 (15.5)	3,745 (15.0)	
Occupation					
Unemployed	36,656 (7.7)	17,585 (6.5)	15,641 (8.6)	3,430 (13.7)	<0.001
Employed	438,157 (91.9)	250,490 (93.1)	166,170 (91.0)	21,497 (85.9)	
Don’t know or prefer not to answer	1,775 (0.4)	963 (0.4)	720 (0.4)	92 (0.4)	
Current smoking	50,303 (10.6)	25,411 (9.4)	21,223 (11.6)	3,669 (14.7)	<0.001
Alcohol intake at least once per week	330,472 (69.3)	192,193 (71.4)	122,082 (66.9)	16,197 (64.7)	<0.001
Physical activity ≥150 min/wk	342,031 (71.8)	197,066 (73.2)	128,379 (70.3)	16,586 (66.3)	<0.001
Chronic kidney disease	12,676 (2.7)	5,368 (2.0)	6,147 (3.4)	1,161 (4.6)	<0.001
Hypertension	264,639 (55.5)	138,399 (51.4)	109,451 (60.0)	16,789 (67.1)	<0.001
Diabetes	29,069 (6.1)	11,239 (4.2)	14,741 (8.1)	3,089 (12.3)	<0.001
Cardiovascular disease	30,698 (6.4)	12,404 (4.6)	14,851 (8.1)	3,443 (13.8)	<0.001
Cancer	49,967 (10.5)	26,671 (9.9)	20,244 (11.1)	3,052 (12.2)	<0.001
Medication for blood pressure	96,850 (20.3)	44,410 (16.5)	44,522 (24.4)	7,918 (31.6)	<0.001
Medication for blood glucose	17,617 (3.7)	6,431 (2.4)	9,126 (5.0)	2060 (8.2)	<0.001
Medication for serum cholesterol	83,144 (17.4)	37,103 (13.8)	38,657 (21.2)	7,384 (29.5)	<0.001
Total sleep duration, h/d	7.2 ± 1.1	7.1 ± 1.0	7.2 ± 1.1	7.7 ± 1.6	<0.001
Insomnia symptoms	133,888 (28.1)	71,766 (26.7)	52,809 (28.9)	9,313 (37.2)	<0.001
Early chronotype	266,085 (55.8)	148,307 (55.1)	103,580 (56.7)	14,198 (56.7)	<0.001
Snoring	164,809 (34.6)	85,753 (31.9)	68,924 (37.8)	10,132 (40.5)	<0.001
Daytime sleepiness	13,130 (2.8)	1,493 (0.6)	7,085 (3.9)	4,552 (18.2)	<0.001
Genetic risk stratification of atrial fibrillation					
Low	160,204 (33.6)	90,827 (33.8)	60,973 (33.4)	8,404 (33.6)	0.042
Moderate	159,398 (33.4)	89,653 (33.3)	61,256 (33.6)	8,489 (33.9)	
High	156,986 (32.9)	88,558 (32.9)	60,302 (33.0)	8,126 (32.5)	

Values are mean ± SD or n (%).

a Group differences tested using analysis of variance or chi-squared test.

As depicted in Supplemental Table 5, compared to never/rarely napping, sometimes and usually napping were associated with a 1.08-fold (HR: 1.08; 95% CI: 1.06-1.11) and 1.17-fold AF risk (HR: 1.17; 95% CI: 1.12-1.22), respectively, independently from AF genetic risk and other covariates. More frequent napping was consistently associated with a higher AF incidence, regardless of the genetic risk stratum (Supplemental Figure 3). Stronger associations were observed among individuals of low and moderate genetic risk (*P* for interaction <0.001).

**Figure 1:**
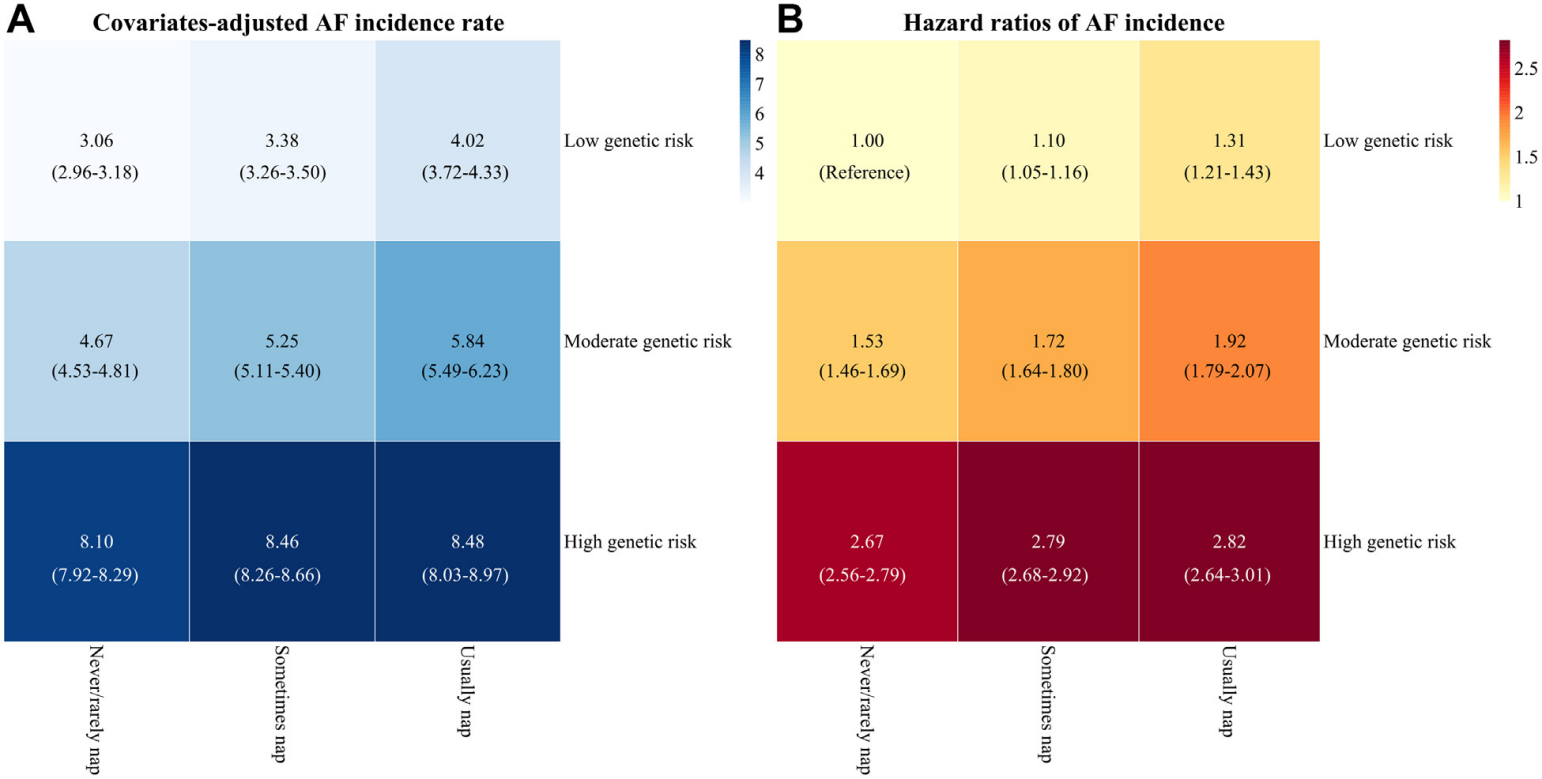
Adjusted Incidence Rate and Hazard Ratios of Atrial Fibrillation According to Combinations of Napping and Genetic Risk Stratification (A) Covariates-adjusted atrial fibrillation incidence rate and 95% CI per 1,000 person-years; (B) HRs and 95% CI of incident atrial fibrillation compared with the combination of never/rarely have a nap and low genetic risk. AF = atrial fibrillation.

The adjusted AF incidence rate and HRs according to combinations of napping and AF genetic risk were summarized in Figure 1. As shown in Figure 1A, individuals who never/rarely have a nap and of low genetic risk had the lowest AF incidence rate of 3.06 per 1,000 person-years. As shown in Figure 1B, compared to individuals never/rarely have a nap and of low genetic risk, individuals both usually have a nap and of high genetic risk had a 182% higher AF risk (HR: 2.82; 95% CI: 2.64-3.01).

**Figure 2:**
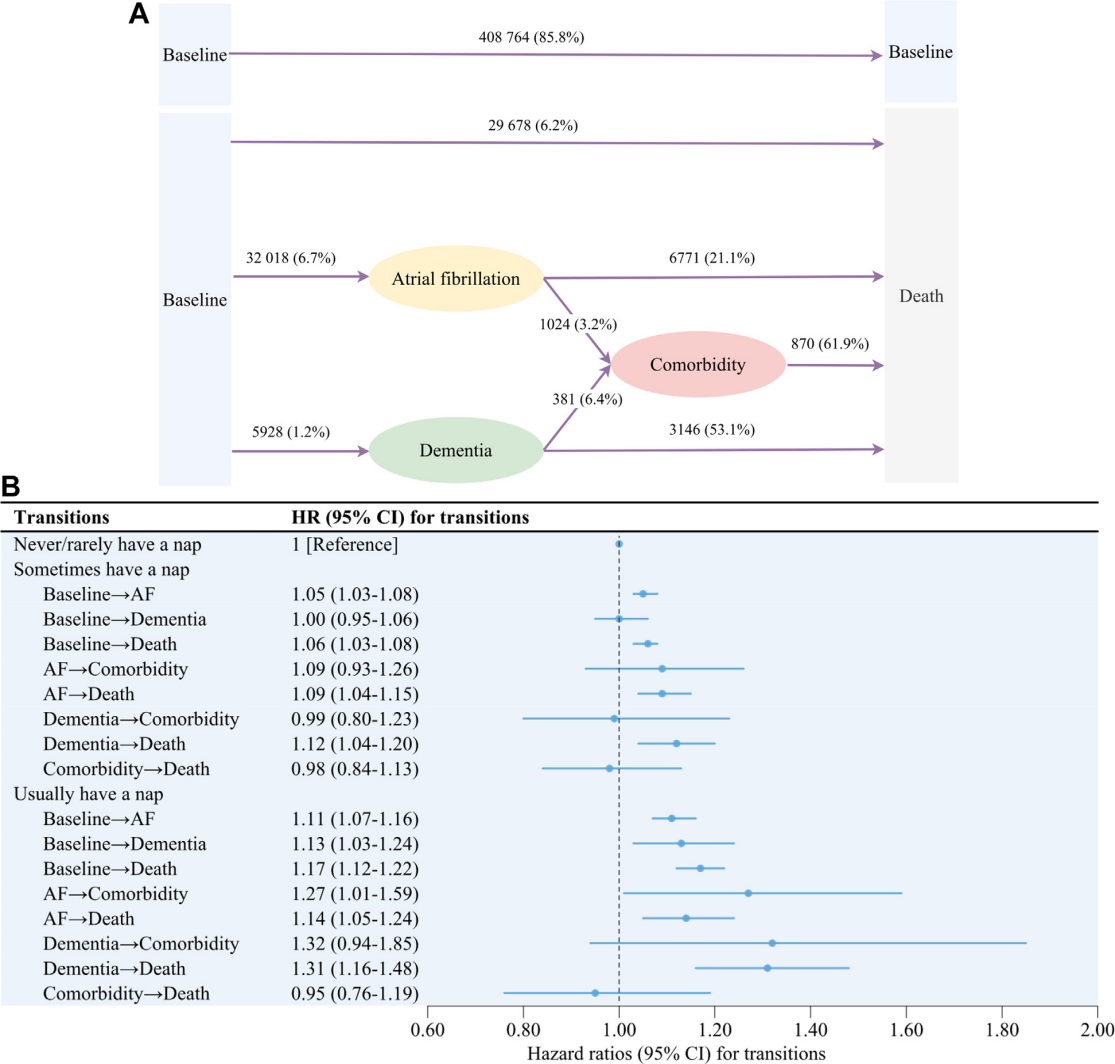
Associations Between Napping and Disease Transition Hazards of Atrial Fibrillation and Dementia (A) Observed dynamic transitions (number and percentage) of atrial fibrillation and dementia. The comorbidity referred to the state of coexistence of incident AF and dementia; (B) HRs of dynamic transitions of AF and dementia according to napping frequency. AF = atrial fibrillation.

As shown in Figure 2A, a total of 408,764 (85.8%) participants remained free of predefined events during a median follow-up of 13.82 years, while 32,018 (6.7%) and 5,928 (1.2%) participants were diagnosed with AF and dementia, respectively. In addition, 1,024 (3.2%) participants with AF were further diagnosed with dementia and 381 (6.4%) participants with dementia were further diagnosed with AF. As shown in Figure 2B, frequent daytime napping was consistently associated with elevated hazards of most transitions, including elevated transition hazards from incident AF to comorbidity of AF and dementia. Usually napping was consistently associated with the worse prognosis of AF, including a 1.27-fold transition risk (HR: 1.27; 95% CI: 1.01-1.59) from AF to comorbidity of AF and dementia, and 1.14-fold transition risk (HR: 1.14; 95% CI: 1.05-1.24) from AF to death (Figure 2B).

**Figure 3:**
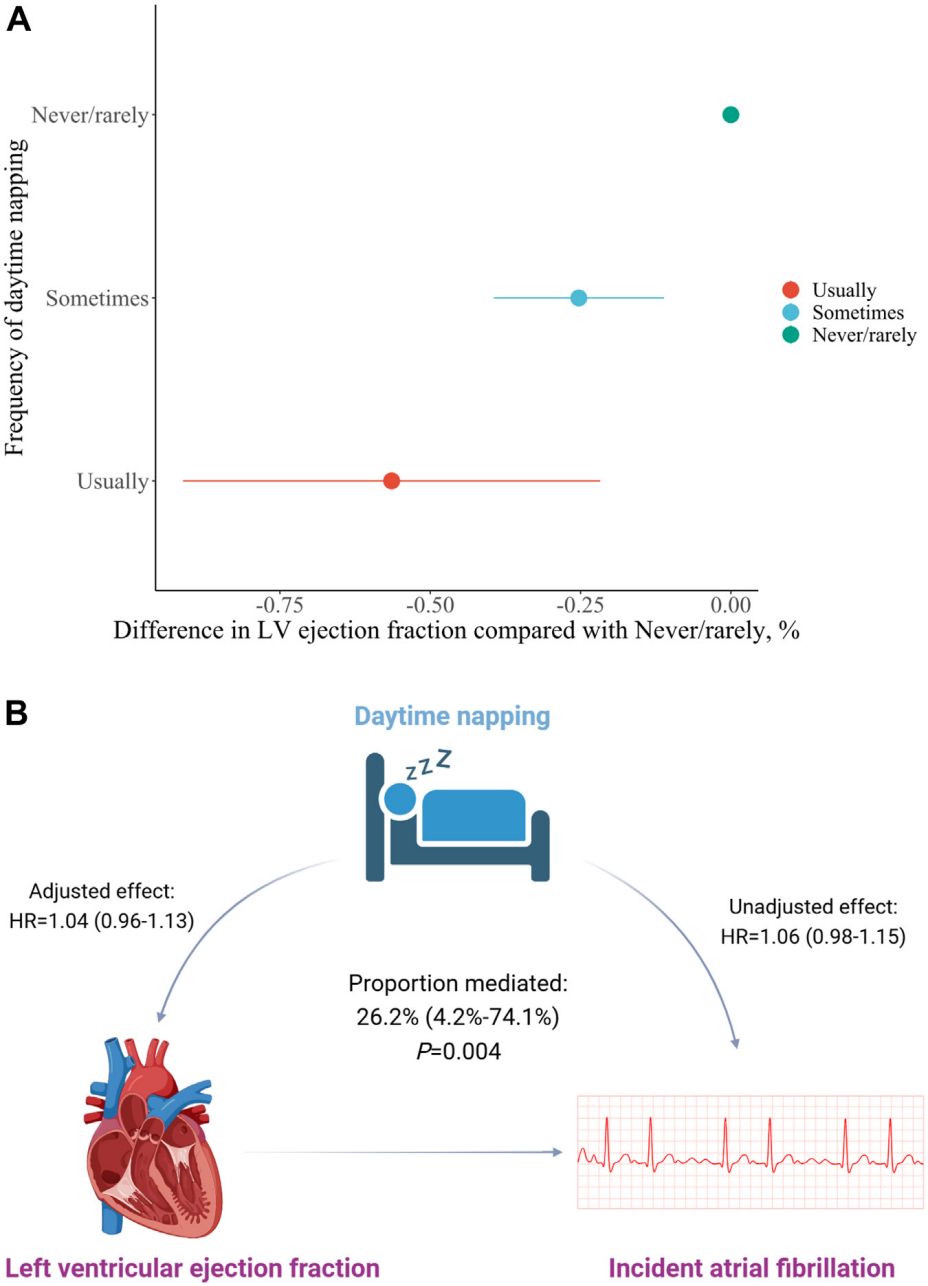
**Associations Between Napping and Left Ventricular Ejection Fraction, and the Underlying Mediation Pathway** (A) Adjusted differences in left ventricular ejection fraction comparing frequency of daytime napping; (B) mediation pathway of left ventricular ejection fraction. LV = left ventricular.

Among evaluated LV size and function indices, only the LV ejection fraction significantly mediated associations between napping and AF incidence (Figure 3). As shown in Figure 3A, compared with the never/rarely group, individuals napping sometimes and usually have significantly lower LV ejection fraction. As shown in Figure 3B, 26.2% (95% CI: 4.2%-74.1%; *P* = 0.004) of observed associations between napping and incident AF was intermediated via LV ejection fraction, while mediation proportions were both small (<3%) and insignificant for LV end diastolic and systolic volumes, LV stroke volume, cardiac output, and cardiac index.

**Figure 4:**
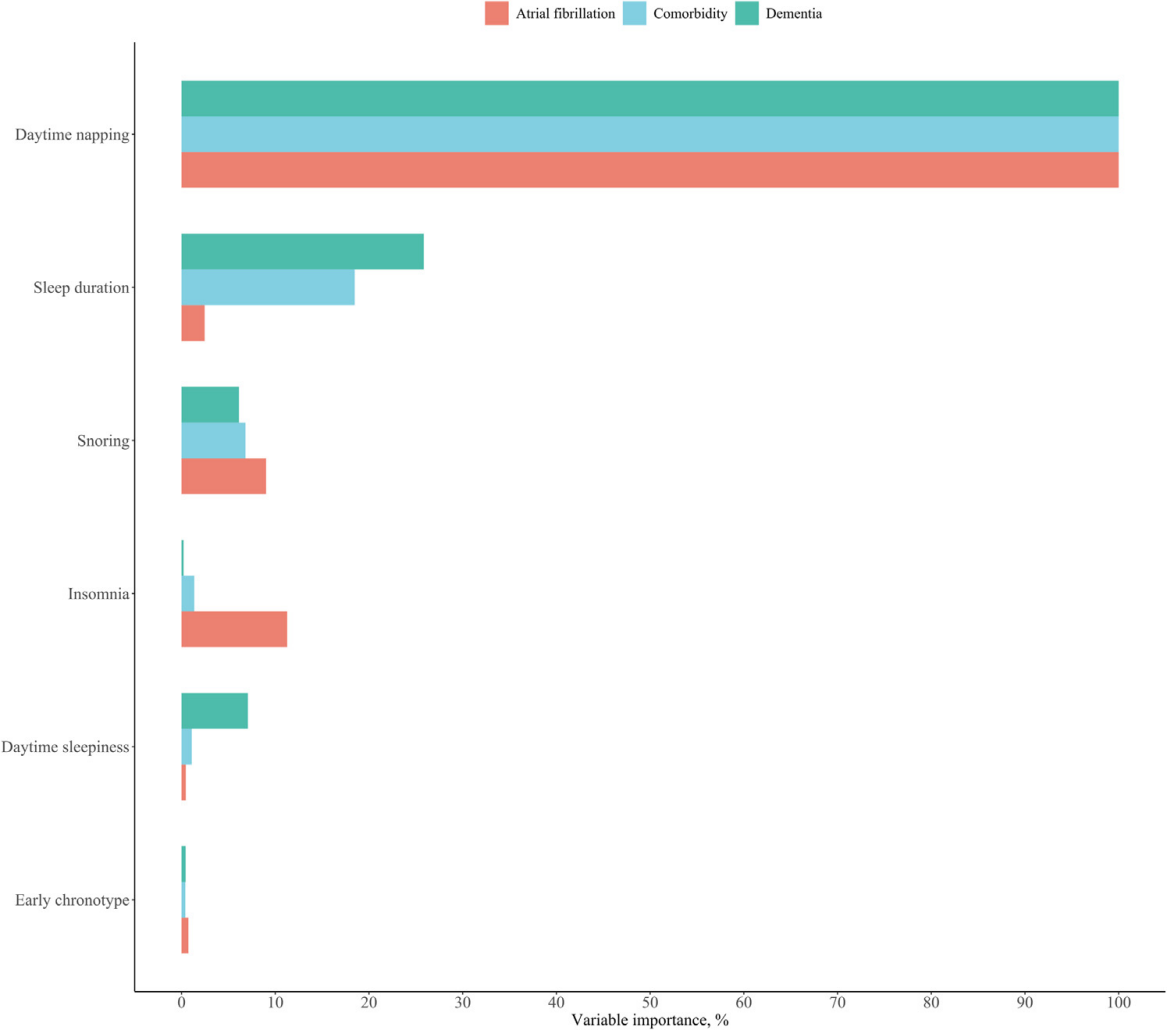
**Relative Importance of Napping and Other Sleep Behaviors in Predicting Incident Atrial Fibrillation, Dementia, and Comorbidity** A permutation-based method was applied to investigate the importance of included variables.

Among all sleep behaviors (Figure 4), daytime napping remained the top of importance for predicting incident AF, dementia, and the comorbidity of AF and dementia, surpassing insomnia, snoring, sleep duration, early chronotype, and daytime sleepiness.

In sensitivity analyses, the magnitude of associations was not materially altered after excluding prevalent cardiovascular conditions (Supplemental Table 6), controlling for other sleep behaviors (Supplemental Tables 7 to 9), excluding the first 2 years of AF cases (Supplemental Table 10), accounting for competing risk from death (Supplemental Table 11), controlling for body mass index (Supplemental Table 12), Fried frailty phenotype (Supplemental Table 13), and night shift work (Supplemental Table 14). After switching to the causal mediation analytical approach, the proportion mediated via LV ejection fraction increased, intermediating 57.7% of observed associations (Supplemental Figure 4). Significant interactions between napping and covariates were observed (Supplemental Figure 5), with stronger associations between napping and AF risk observed among individuals aged <60 years, women, and those drinking less than once per week. Excluding individuals developing dementia within 2 years of incident AF or controlling for snoring also did not materially alter the results (Supplemental Figures 6 and 7). Nonresponse analysis (Supplemental Table 15) showed that excluded participants, compared to included participants, were older, more likely to be men, non-White ethnicity, unemployed, less educated, had lower income, and more prevalent chronic diseases.

## Discussion

As shown in the Central Illustration, the current study observed associations of daytime napping with both AF onset and dynamic transitions with dementia. The associations remained after controlling for other known major risk factors of AF, including body mass index and other sleep behaviors. Notably, we found usually napping was also associated with the transition from incident AF to comorbidity of AF and dementia. These novel findings highlight the necessity of screening for frequent napping for fulfilling both AF primary prevention and improving long-term prognosis, in conjunction with delaying dementia onset. Finally, LV ejection fraction was identified as the main operating mechanism linking daytime napping and AF risk. To the best of our knowledge, the current study is the first one simultaneously investigating the associations between daytime napping, AF genetic predisposition, and incident AF, as well as the dynamic disease transitions of AF and dementia.

**Central Illustration:**
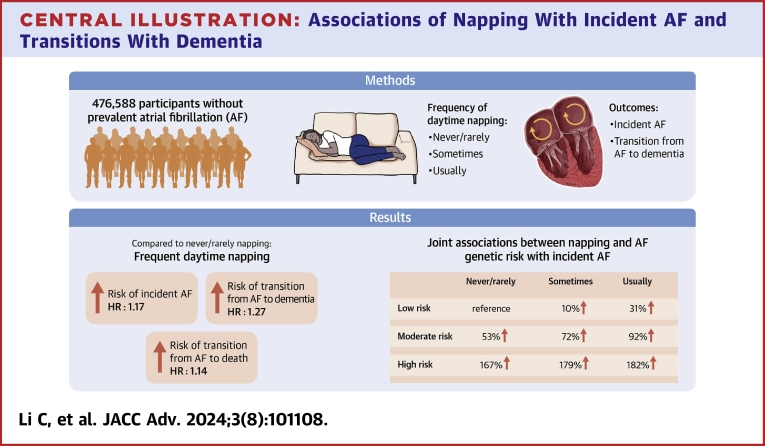
Associations of Napping With Incident AF and Transitions With Dementia Compared with never/rarely napping, frequent napping was associated with both AF risk and transitions with dementia. Joint associations were observed between napping and AF genetic predisposition, with the highest risk observed among those combining frequent napping and high genetic risk. AF = atrial fibrillation.

Despite the enriched and widespread recognition regarding the cardiovascular health impact of sleep, prospective studies investigating daytime napping in relation to AF risk are scarce. A previous study systematically evaluated associations between adherence to a healthy sleep pattern and incident AF risk, and found that better sleep health was consistently associated with a lower AF risk, independently from the genetic predisposition.^11^ The researchers did not account for the frequency of daytime napping. In another cross-sectional study, researchers found habitual daytime napping was associated with 28% higher odds of prevalent AF.^23^ So far, few prospective studies have investigated associations between napping and AF incidence. Several recent studies have reported positive associations between napping and dementia incidence.^14^^,^^15^ Nevertheless, no studies have accounted for the dynamic transitions of AF and dementia when evaluating the role of daytime napping, leaving important research gap and preventing further efforts targeting primary prevention of the 2 diseases. Notably, the observed frequent napping behavior could be an indicator of sleep apnea, along with daytime sleepiness. It has been found that sleep apnea could account for the excessive cardiovascular disease risk among habitual nappers.^32^ Therefore, our findings support frequent napping as a valuable characteristic of at-risk population, with sleep apnea serving as dominant mechanism.

Compared with previous studies, our study additionally considered dynamic disease transitions of AF and dementia, providing important implications regarding the onset and prognosis of the 2 diseases. With the multistate analytical approach, we found that frequent daytime napping was consistently associated with elevated transition hazards from baseline to incident AF or dementia, which was in line with our results from Cox regression and previous studies using traditional analytical approach.^14^ Intriguingly, we found, for the very first time, that daytime napping was also associated with elevated transition hazards from AF to comorbidity of AF and dementia. On one hand, accumulating evidence has indicated that individuals living with AF tend to have elevated risks of cognitive impairment and dementia.^33^, ^34^, ^35^ On the other hand, people living with the comorbidity of AF and dementia constitute a special population, with more prevalent under-prescription of oral anticoagulant and higher long-term mortality risk.^36^^,^^37^ Hence, preventing transition from AF to the comorbidity of AF and dementia is of huge significance, highlighting the significance of our findings to support the potential importance of screening for napping in view of the association with incident AF and dementia.

We identified reduced LV ejection fraction as one operating mechanism linking napping to AF. This was noteworthy. According to recent guidelines, LV ejection fraction remains the major parameter for diagnosis, phenotyping, prognosis, and treatment decisions of AF.^17^ Intriguingly, a previous study also found that excessive daytime napping was associated with an elevated heart failure incidence, independently from other sleep behaviors.^12^ Therefore, these findings also support that reduced LV ejection could serve as the shared operating mechanism linking napping to both AF and heart failure. More attention could be warranted to the monitoring of LV ejection fraction among habitual nappers to prevent AF onset and transition at the early presymptomatic stage. Notably, we observed slightly attenuated associations after further adjustment of body mass index and physical frailty. In addition, the left atrium size and function were regarded as important parameters of AF,^17^ which we could examine the mediation role, due to data restrictions. Therefore, further investigations are warranted to examine other potential mediators linking napping and AF.

Our study possesses several strengths. First, with the large sample size and long-term follow-up of the UK Biobank, we were able to examine the prospective and joint associations between daytime napping and AF genetic predisposition with AF incidence. Second, with the multistate models, we were able to comprehensively evaluate the dynamic transitions of AF and dementia, and evaluate the role of napping in these transitions. Third, we incorporated LV ejection fraction into evaluations, providing evidence of the underlying operating mechanism linking napping with AF incidence. Finally, various sensitivity analyses were conducted, supporting the robustness of major findings, including further controlling for other sleep behaviors.

### Study Limitations

We also acknowledge important limitations. First, self-reported data were used to evaluate napping and other sleep behaviors. In addition, only the frequency of daytime napping was evaluated. Further studies with objective and comprehensive napping measurements are therefore warranted to confirm our findings. Second, as a temporal behavior, longitudinal repeated measurements of napping were not evaluated. Therefore, we could not examine the long-term change in napping and the potential impact of the longitudinal change on AF risk, limiting the implications of the study. Third, most of the UK Biobank participants were of White ethnicity, hence restricting the generalization of our findings. Fourth, significant differences in baseline characteristics were observed between included and excluded participants, indicating the potential selection bias. Fifth, caution should be taken when interpreting mediation analysis results, as large number of participants were excluded. Sixth, the reported 95% CI was not adjusted for multiple comparisons, necessitating cautious interpretation of findings. Finally, as an observational study, we could not preclude the impact of residual confounding.

## Conclusions

We found that frequent daytime napping was both associated with elevated AF incidence and dynamic transitions of AF and dementia, including transition from incident AF to comorbidity of AF and dementia. Reduced LV ejection fraction was identified as one operating mechanism linking napping and AF risk. These findings highlight the importance of screening for napping in view of the association with incident AF and dementia. And routine evaluations of LV ejection fraction could be warranted to timely identify early indications of AF onset among habitual nappers.

PERSPECTIVES **COMPETENCY IN PATIENT CARE:** Frequent daytime napping was not only associated with elevated AF risk, but the transition from AF to comorbidity of AF and dementia. Reduced LV ejection fraction was identified as one operating mechanism linking napping and AF risk.

**TRANSLATIONAL OUTLOOK:** Further investigations are warranted to examine the benefits of screening for frequent napping for fulfilling both AF primary prevention and delaying dementia onset. And routine evaluations of LV ejection fraction could be warranted to timely identify early indications of AF onset among habitual nappers.

## Funding support and author disclosures

This study was supported by grants from the 10.13039/501100012166National Key Research and Development Program of China (No. 2022YFF1203001), 10.13039/501100001809National Natural Science Foundation of China (No. 72125009), Chinese Scientific and Technical Innovation Project 2030 (No. 2018AAA0102100), 10.13039/501100003345CAMS Innovation Fund for Medical Sciences (No. 2019-I2M-5-046), and 10.13039/501100007937PKU-Baidu Fund (No. 2020BD004, 2020BD005). The authors have reported that they have no relationships relevant to the contents of this paper to disclose.
