# Supplementary material for: Daytime Napping, Incident Atrial Fibrillation, and Dynamic Transitions With Dementia
Source: JACC Adv. 2024 Jul 12;3(8):101108. doi: 10.1016/j.jacadv.2024.101108 (PMC11299576; doi:10.1016/j.jacadv.2024.101108)
Supplement: Supplementary data [file mmc1.docx]

**Supplemental Table 1.** Definition and assessment of atrial fibrillation in UK Biobank.

| **Approach** | **Assessment** | **UK Biobank Data-Field ID** |
| --- | --- | --- |
| ICD-10 codes (incorporating data from inpatient records and death registry) | Codes I48, I48.1, I48.2, I48.3, I48.4, I48.9 | 131350, 131351 |
| OPCS4 codes | Codes K50.1, K62.2, K62.3, K62.4 | 41272, 41282 |
| Self-report of history of cardiovascular disease | Verbal interview for non-cancer illness | 20002 |

**Supplemental Table 2.** ICD-10 codes used to ascertain all-cause dementia cases.

| ICD-10 codes | ICD-10 text | All-cause dementia |
| --- | --- | --- |
| A81.0 | Sporadic Creutzfeldt-Jakob disease | √ |
| F00 | Dementia in Alzheimer's disease | √ |
| F00.0 | Dementia in Alzheimer's disease with early onset | √ |
| F00.1 | Dementia in Alzheimer's disease with late onset | √ |
| F00.2 | Dementia in Alzheimer's disease, atypical or mixed type | √ |
| F00.9 | Dementia in Alzheimer's disease, unspecified | √ |
| F01 | Vascular dementia | √ |
| F01.0 | Vascular dementia of acute onset | √ |
| F01.1 | Multi-infarct dementia | √ |
| F01.2 | Subcortical vascular dementia | √ |
| F01.3 | Mixed cortical and sub-cortical vascular dementia | √ |
| F01.8 | Other vascular dementia | √ |
| F01.9 | Vascular dementia, unspecified | √ |
| F02 | Dementia in other diseases classified elsewhere | √ |
| F02.0 | Dementia in Picks disease | √ |
| F02.1 | Dementia in Creutzfeldt-Jacob disease | √ |
| F02.2 | Dementia in Huntington’s disease | √ |
| F02.3 | Dementia in Parkinson’s disease | √ |
| F02.4 | Dementia in HIV disease | √ |
| F02.8 | Dementia in other specified diseases classified elsewhere | √ |
| F03 | Unspecified dementia | √ |
| F05.1 | Delirium superimposed on dementia | √ |
| F10.6 | Mental and behavioural disorders due to use of alcohol - amnesic syndrome | √ |
| G30 | Alzheimer’s disease | √ |
| G30.0 | Alzheimer’s disease with early onset | √ |
| G30.1 | Alzheimer’s disease with late onset | √ |
| G30.8 | Other Alzheimer's disease | √ |
| G30.9 | Alzheimer's disease unspecified | √ |
| G31.0 | Circumscribed brain atrophy | √ |
| G31.1 | Senile degeneration of brain | √ |
| G31.8 | Other specified degenerative diseases of  nervous system | √ |
| I67.3 | Binswanger’s disease |  |

**Supplemental Table 3.** Information of genetic variants associated with atrial fibrillation in UK Biobank, applied for constructing the genetic risk score.

| SNP | Chr | Risk/Ref allele | RR | p-value | Nearest Gene (s) |
| --- | --- | --- | --- | --- | --- |
| rs187585530 | 1 | A/G | 1.55 | 1.18E-10 | UBE4B |
| rs880315 | 1 | C/T | 1.04 | 5.04E-09 | CASZ1 |
| rs7529220 | 1 | C/T | 1.04 | 0.0001002 | HSPG2,CELA3B |
| rs2885697 | 1 | G/T | 1.04 | 1.77E-07 | SCMH1 |
| rs11590635 | 1 | A/G | 1.12 | 5.28E-05 | AGBL4 |
| rs146518726 | 1 | A/G | 1.18 | 2.05E-10 | C1orf185 |
| rs12044963 | 1 | T/G | 1.08 | 1.61E-12 | KCND3 |
| rs4484922 | 1 | G/C | 1.07 | 4.57E-16 | CASQ2 |
| rs79187193 | 1 | G/A | 1.12 | 8.07E-10 | GJA5 |
| rs11264280 | 1 | T/C | 1.14 | 4.60E-59 | KCNN3,PMVK |
| rs72700114 | 1 | C/G | 1.22 | 7.32E-48 | METTL11B,LINC01142 |
| rs608930 | 1 | G/T | 1.1 | 1.94E-42 | GORAB,PRRX1 |
| rs10753933 | 1 | T/G | 1.08 | 5.83E-25 | PPFIA4 |
| rs4951261 | 1 | C/A | 1.05 | 1.17E-09 | NUCKS1 |
| rs6546620 | 2 | C/T | 1.07 | 2.96E-14 | KIF3C |
| rs6742276 | 2 | A/G | 1.05 | 2.42E-11 | XPO1 |
| rs2540949 | 2 | A/T | 1.08 | 8.17E-25 | CEP68 |
| rs10165883 | 2 | C/T | 1.07 | 5.83E-19 | SNRNP27 |
| rs72926475 | 2 | G/A | 1.07 | 3.49E-10 | REEP1,KDM3A |
| rs28387148 | 2 | T/C | 1.06 | 7.35E-06 | GYPC |
| rs67969609 | 2 | G/C | 1.06 | 5.10E-06 | TEX41 |
| rs12992412 | 2 | T/A | 1.04 | 2.30E-08 | MBD5 |
| rs56181519 | 2 | C/T | 1.08 | 1.52E-19 | WIPF1,CHRNA1 |
| rs35504893 | 2 | T/C | 1.09 | 6.89E-25 | TTN |
| rs295114 | 2 | C/T | 1.07 | 1.76E-20 | SPATS2L |
| rs35544454 | 2 | A/T | 1.05 | 1.53E-06 | ERBB4 |
| rs6810325 | 3 | C/T | 1.08 | 5.24E-23 | CAND2 |
| rs73032363 | 3 | A/G | 1.04 | 3.59E-08 | THRB |
| rs6790396 | 3 | G/C | 1.07 | 4.13E-18 | SCN10A |
| rs2306272 | 3 | C/T | 1.05 | 4.54E-11 | LRIG1 |
| rs17005647 | 3 | T/C | 1.03 | 0.0001942 | FRMD4B |
| rs7632427 | 3 | T/C | 1.04 | 1.10E-08 | EPHA3 |
| rs17490701 | 3 | G/A | 1.07 | 5.43E-11 | PHLDB2 |
| rs1278493 | 3 | G/A | 1.03 | 1.59E-05 | PPP2R3A |
| rs4855075 | 3 | T/C | 1.06 | 4.00E-09 | GNB4 |
| rs60902112 | 3 | T/C | 1.04 | 6.09E-07 | XXYLT1 |
| rs9872035 | 3 | C/T | 1.04 | 1.80E-08 | PAK2 |
| rs3822259 | 4 | T/G | 1.05 | 1.93E-09 | WDR1 |
| rs1458038 | 4 | T/C | 1.04 | 6.79E-06 | PRDM8,FGF5 |
| rs3960788 | 4 | C/T | 1.05 | 2.09E-12 | SLC9B1 |
| rs2129977 | 4 | A/G | 1.49 | 3.09e-525 | PITX2,C4orf32 |
| rs55754224 | 4 | T/C | 1.05 | 9.25E-09 | CAMK2D |
| rs10213171 | 4 | G/C | 1.11 | 6.09E-14 | ARHGAP10 |
| rs10520260 | 4 | A/G | 1.06 | 8.98E-12 | HAND2 |
| rs6596717 | 5 | C/A | 1.03 | 3.22E-06 | LOC102467213,EFNA5 |
| rs716845 | 5 | A/G | 1.06 | 1.16E-13 | KCNN2 |
| rs2012809 | 5 | G/A | 1.05 | 1.98E-07 | FBN2,SLC27A6 |
| rs34750263 | 5 | T/C | 1.09 | 2.89E-30 | WNT8A,NME5 |
| rs174048 | 5 | C/T | 1.07 | 1.05E-11 | ARHGAP26,NR3C1 |
| rs12188351 | 5 | A/G | 1.07 | 1.94E-05 | SLIT3 |
| rs6882776 | 5 | G/A | 1.06 | 3.18E-14 | NKX2-5 |
| rs73366713 | 6 | G/A | 1.11 | 5.80E-21 | ATXN1 |
| rs34969716 | 6 | A/G | 1.09 | 2.91E-25 | KDM1B |
| rs1307274 | 6 | T/G | 1.08 | 3.85E-08 | C6orf1,NUDT3 |
| rs3176326 | 6 | G/A | 1.06 | 7.95E-11 | CDKN1A |
| rs6907805 | 6 | G/T | 1.04 | 1.10E-08 | CGA,ZNF292 |
| rs210632 | 6 | A/G | 1.05 | 2.75E-08 | GOPC |
| rs17079881 | 6 | G/A | 1.09 | 4.23E-16 | SLC35F1 |
| rs13191450 | 6 | A/C | 1.07 | 8.92E-21 | GJA1,HSF2 |
| rs12208899 | 6 | A/G | 1.05 | 1.95E-08 | LINC00326,EYA4 |
| rs117984853 | 6 | T/G | 1.12 | 8.38E-17 | UST |
| rs11768850 | 7 | T/C | 1.04 | 4.96E-08 | SUN1 |
| rs55734480 | 7 | A/G | 1.05 | 7.34E-10 | DGKB |
| rs6462078 | 7 | A/C | 1.06 | 1.35E-11 | CREB5 |
| rs74910854 | 7 | G/A | 1.1 | 3.36E-09 | GTF2I |
| rs11773884 | 7 | A/G | 1.05 | 4.72E-09 | CDK6 |
| rs62483627 | 7 | A/G | 1.05 | 5.17E-09 | COG5 |
| rs11773845 | 7 | A/C | 1.12 | 4.61E-58 | CAV1 |
| rs55985730 | 7 | G/T | 1.1 | 1.81E-08 | OPN1SW |
| rs7789146 | 7 | G/A | 1.06 | 6.51E-10 | KCNH2 |
| rs35620480 | 8 | C/A | 1.05 | 1.01E-06 | LINC00208,GATA4 |
| rs7508 | 8 | A/G | 1.07 | 2.22E-19 | ASAH1 |
| rs7846485 | 8 | C/A | 1.09 | 3.71E-15 | XPO7 |
| rs62521286 | 8 | G/A | 1.13 | 1.24E-16 | FBXO32 |
| rs35006907 | 8 | A/C | 1.05 | 2.76E-09 | MTSS1,LINC00964 |
| rs7460121 | 8 | A/G | 1.07 | 1.65E-08 | MIR30B |
| rs6993266 | 8 | A/G | 1.05 | 9.73E-10 | PTK2 |
| rs4977397 | 9 | A/G | 1.04 | 8.60E-09 | SLC24A2,MLLT3 |
| rs4385527 | 9 | A/G | 1.1 | 2.26E-36 | C9orf3 |
| rs4743034 | 9 | A/G | 1.05 | 3.98E-09 | ZNF462 |
| rs10760361 | 9 | G/T | 1.04 | 7.03E-09 | PSMB7 |
| rs2274115 | 9 | G/A | 1.04 | 1.78E-05 | LHX3 |
| rs7919685 | 10 | G/T | 1.06 | 5.00E-16 | REEP3 |
| rs7096385 | 10 | T/C | 1.06 | 1.52E-05 | SIRT1 |
| rs60212594 | 10 | G/C | 1.12 | 6.48E-27 | SYNPO2L |
| rs11001667 | 10 | G/A | 1.06 | 1.06E-11 | C10orf11 |
| rs1044258 | 10 | T/C | 1.05 | 1.07E-09 | C10orf76 |
| rs11598047 | 10 | G/A | 1.17 | 4.83E-58 | NEURL |
| rs10749053 | 10 | T/C | 1.06 | 1.84E-07 | RBM20 |
| rs1822273 | 11 | G/A | 1.07 | 8.99E-17 | NAV2 |
| rs949078 | 11 | C/T | 1.05 | 4.77E-11 | SORL1,MIR100HG |
| rs76097649 | 11 | A/G | 1.13 | 2.19E-20 | KCNJ5 |
| rs10842383 | 12 | C/T | 1.11 | 1.02E-25 | LINC00477,BCAT1 |
| rs113819537 | 12 | C/G | 1.05 | 2.23E-09 | SSPN |
| rs12809354 | 12 | C/T | 1.08 | 5.48E-16 | PKP2 |
| rs7978685 | 12 | T/C | 1.06 | 5.99E-12 | NACA |
| rs35349325 | 12 | T/C | 1.05 | 9.04E-13 | BEST3 |
| rs11180703 | 12 | G/A | 1.05 | 3.58E-10 | KRR1,PHLDA1 |
| rs883079 | 12 | T/C | 1.13 | 1.26E-51 | TBX5 |
| rs12810346 | 12 | T/C | 1.07 | 2.34E-09 | TBX5-AS1,TBX3 |
| rs10773657 | 12 | C/A | 1.06 | 4.22E-07 | HIP1R |
| rs12298484 | 12 | C/T | 1.05 | 2.05E-09 | DNAH10 |
| rs6560886 | 12 | C/T | 1.04 | 3.13E-05 | FBRSL1 |
| rs9580438 | 13 | C/T | 1.06 | 1.01E-13 | LINC00540,BASP1P1 |
| rs35569628 | 13 | T/C | 1.04 | 3.00E-05 | CUL4A |
| rs28631169 | 14 | T/C | 1.07 | 3.80E-14 | MYH7 |
| rs2145587 | 14 | A/G | 1.08 | 2.32E-21 | AKAP6 |
| rs73241997 | 14 | T/C | 1.07 | 1.10E-13 | SNX6,CFL2 |
| rs2738413 | 14 | A/G | 1.08 | 1.81E-29 | SYNE2 |
| rs74884082 | 14 | C/T | 1.05 | 7.26E-08 | DPF3 |
| rs10873299 | 14 | A/G | 1.05 | 9.62E-11 | LRRC74,IRF2BPL |
| rs147301839 | 15 | C/A | 1.6 | 0.02416 | GCOM1 |
| rs62011291 | 15 | G/A | 1.05 | 6.14E-09 | USP3 |
| rs12591736 | 15 | G/A | 1.06 | 2.47E-09 | TLE3,UACA |
| rs74022964 | 15 | T/C | 1.11 | 1.27E-27 | HCN4,REC114 |
| rs12908004 | 15 | G/A | 1.08 | 1.95E-14 | LINC00927,ARNT2 |
| rs12908437 | 15 | T/C | 1.05 | 1.25E-10 | IGF1R |
| rs2286466 | 16 | G/A | 1.07 | 3.53E-14 | RPS2 |
| rs2359171 | 16 | A/T | 1.21 | 2.94E-100 | ZFHX3 |
| rs7225165 | 17 | G/A | 1.05 | 2.41E-05 | YWHAE,CRK |
| rs8073937 | 17 | G/A | 1.05 | 1.02E-11 | POLR2A,TNFSF12 |
| rs72811294 | 17 | G/C | 1.07 | 6.87E-09 | MYOCD |
| rs11658278 | 17 | T/C | 1.03 | 3.17E-06 | ZPBP2 |
| rs242557 | 17 | G/A | 1.04 | 4.35E-09 | MAPT |
| rs76774446 | 17 | A/C | 1.07 | 1.13E-08 | GOSR2 |
| rs7219869 | 17 | G/C | 1.05 | 1.49E-10 | KCNJ2,CASC17 |
| rs12604076 | 17 | T/C | 1.02 | 0.001278 | CYTH1 |
| rs9953366 | 18 | C/T | 1.05 | 9.03E-11 | SMAD7 |
| rs8088085 | 18 | A/C | 1.03 | 5.15E-06 | MEX3C |
| rs2145274 | 20 | A/C | 1.11 | 6.97E-13 | CASC20,BMP2 |
| rs7269123 | 20 | C/T | 1.05 | 5.59E-09 | C20orf166 |
| rs2834618 | 21 | T/G | 1.12 | 2.93E-18 | LOC100506385 |
| rs465276 | 22 | G/A | 1.05 | 1.84E-11 | TUBA8 |
| rs133902 | 22 | T/C | 1.04 | 1.06E-07 | MYO18B |

SNP: single nucleotide polymorphism; Chr: Chromosome; RR: relative risk

**Supplemental Table 4.** Covariates definition and assessment in UK Biobank.

| **Covariates** | **Definition** | **Assessment** | **UK Biobank** **Data-Field ID** |
| --- | --- | --- | --- |
| Age (years) | Age in years | Difference between date attended baseline assessment and date of birth recorded by NHS | 21003 |
| Sex | Men, Women | NHS derived and/or touchscreen questionnaire | 31 |
| Ethnic background | White, Non-White (Mixed, Asian, Black, Chinese, Other) | Touchscreen questionnaire: “What is your ethnic group?” | 21000 |
| Education attainment | Categorized into higher education (college or university degree, other professional qualifications), secondary education (A levels, AS levels or equivalent, O levels/GCSEs or equivalent, CSEs or equivalent, NVQ or HND or HNC or equivalent), and lower education (none of the above). | Touchscreen questionnaire: “Which of the following qualifications do you have? (You can select more than one)” | 6138 |
| Annual household income, £ | The total household income (£) before tax, categorized into: <18 000, 18 000-30 999, 31 000-51 999, 52 000-100 000, and >100 000. | Touchscreen questionnaire: “What is the average total income before tax received by your HOUSEHOLD?” | 738 |
| Occupation | Categorized into two levels, including employed (in paid employment or self-employed, retired, doing unpaid or voluntary work, or being full or part-time students) and unemployed. | Touchscreen questionnaire: “Which of the following describes your current situation? (You can select more than one answer)” | 6142, 20119 |
| Alcohol intake | At least once per week, less than once per week | Touchscreen questionnaire: “About how often do you drink alcohol?” | 1558 |
| Current smoking | Yes, No | Current smoking status summary | 20116 |
| Moderate-to-vigorous physical activity ≥ 150 minutes per week | Yes (moderate physical activity ≥ 150 or vigorous physical activity ≥ 75 or combined duration ≥ 150, with 1 minute of vigorous activity equaling 2 minutes of moderate activity), No | Metabolic Equivalent Task minutes of moderate and vigorous physical activity per week;  Self-reported duration of conducting moderate and vigorous physical activity per day, transformed into per week. | 22038, 22039, 894, 914 |
| Hypertension | Yes, No | Touchscreen questionnaire and verbal interview: self-reported hypertension or anti-hypertensive medication use;  Average SBP/DBP ≥ 140/90 mm Hg at baseline;  First occurrence of diabetes recorded using ICD-10 (I10-I15) | 6150, 20002, 20003, 6153, 6177, 4079, 4080, 93, 94, 131286- 131295 |
| Diabetes | Yes, No | Touchscreen questionnaire and verbal interview: self-reported diabetes (diabetes, type 1 diabetes or type 2 diabetes) or medication use for lowering blood glucose;  Plasma hba1c ≥ 48 mmol/mol (6.5%);  First occurrence of diabetes recorded using ICD-10 (E10-E14) | 2443, 20002, 20003, 6153, 6177, 30750, 130706-130715 |
| Chronic kidney disease | Yes, No | Touchscreen questionnaire and verbal interview: self-reported history of renal/kidney failure;  Creatinine estimated glomerular filtration rate (using the 2021 CKD-EPI equation) < 60 mL/min/1.73 m^2^;  Urinary Albumin to Creatinine ratio ≥ 300mg/g;  First occurrence of chronic renal failure recorded using ICD-10 (N18-N19) | 20002, 31, 30700, 21022, 30500, 30510, 132032-132035 |
| Cancer | Yes, No | Self-reported cancer diagnosis;  Cancer register data (date of cancer diagnosis) | 2453, 40005 |
| Cardiovascular disease | Yes, No | Touchscreen questionnaire and verbal interview: self-reported history of cardiovascular diseases;  First occurrence of stroke recorded using ICD-10 (I60-I64, I69);  First occurrence of coronary heart disease recorded using ICD-10 (I20-I24);  First occurrence of heart failure recorded using ICD-10 (I50);  Coronary heart disease defined using operative procedures (OPCS4 codes K40-K46, K49-K50, and K75);  Atrial fibrillation defined using operative procedures (OPCS4 codes K50.1 and K62.2–K62.4) | 6150, 20002, 131360-131379, 131296-131305, 131354-131355 |
| Medication use | Yes, no | Self-reported medication use for hypertension (blood pressure), diabetes (blood glucose), or serum cholesterol | 20003, 6177, 6153 |

**Supplemental Table 5.** Overall associations between daytime napping and genetic risk stratification with incident atrial fibrillation, controlling for each other.

| **Exposures** | **Events/total** | **Hazard ratio (95% CI) ^*^** | ***P* value** |
| --- | --- | --- | --- |
| Daytime napping | | | |
| Never/rarely | 14 837/269 038 | 1 (Reference) | NA |
| Sometimes | 14 752/182 531 | 1.08 (1.06-1.11) | <0.001 |
| Usually | 2835/25 019 | 1.17 (1.12-1.22) | <0.001 |
| Linear trend | 32 424/476 588 | 1.08 (1.06-1.10) | <0.001 |
| Genetic risk stratification | | | |
| Low | 6593/160 204 | 1 (Reference) | NA |
| Moderate | 10 042/159 398 | 1.54 (1.49-1.59) | <0.001 |
| High | 15 789/156 986 | 2.56 (2.49-2.63) | <0.001 |
| Linear trend | 32 424/476 588 | 1.61 (1.59-1.63) | <0.001 |

CI, confidence interval; NA, not applicable.

^*^ Hazard ratio was derived using Cox proportional hazard regression, which controlled for age, sex, ethnic background, education attainment, family income, employment status, alcohol consumption, physical activity, current smoking, history of chronic kidney disease, hypertension, diabetes, cancer, cardiovascular disease (other than atrial fibrillation), and use of medications to lower blood pressure, glucose, as well as serum cholesterol.

**Supplemental Table 6.** Associations between daytime napping and incident atrial fibrillation, excluding individuals with prevalent cardiovascular disease.

| **Exposures** | **Events/total** | **Hazard ratio (95% CI) ^a^** | ***P* value** |
| --- | --- | --- | --- |
| Daytime napping | | | |
| Never/rarely | 12 898/256 634 | 1 (Reference) | NA |
| Sometimes | 120 28/167 680 | 1.09 (1.06-1.12) | <0.001 |
| Usually | 2102/21 576 | 1.18 (1.13-1.24) | <0.001 |
| Linear trend | 27 028/445 890 | 1.09 (1.07-1.11) | <0.001 |

CI, confidence interval; NA, not applicable.

^a^ Hazard ratio was derived using Cox proportional hazard regression, which controlled for age, sex, ethnic background, education attainment, family income, employment status, alcohol consumption, physical activity, current smoking, history of chronic kidney disease, hypertension, diabetes, cancer, and use of medications to lower blood pressure, glucose, as well as serum cholesterol.

**Supplemental Table 7.** Associations between daytime napping and incident atrial fibrillation, simultaneously controlling for daytime sleepiness.

| **Exposures** | **Hazard ratio (95% CI) ^a^** | ***P* value** |
| --- | --- | --- |
| Daytime napping |  |  |
| Never/rarely | 1 (Reference) | NA |
| Sometimes | 1.08 (1.05-1.10) | <0.001 |
| Usually | 1.14 (1.09-1.19) | <0.001 |
| Daytime sleepiness |  |  |
| No | 1 (Reference) | NA |
| Yes | 1.14 (1.08-1.21) | <0.001 |

CI, confidence interval; NA, not applicable.

^a^ Hazard ratio was derived using Cox proportional hazard regression, which controlled for age, sex, ethnic background, education attainment, family income, employment status, alcohol consumption, physical activity, current smoking, history of chronic kidney disease, hypertension, diabetes, cancer, cardiovascular disease, and use of medications to lower blood pressure, glucose, as well as serum cholesterol. Daytime sleepiness was also adjusted.

**Supplemental Table 8.** Associations between daytime napping and incident atrial fibrillation, simultaneously controlling for total sleep duration.

| **Exposures** | **Hazard ratio (95% CI) ^a^** | ***P* value** |
| --- | --- | --- |
| Daytime napping |  |  |
| Never/rarely | 1 (Reference) | NA |
| Sometimes | 1.08 (1.06-1.11) | <0.001 |
| Usually | 1.18 (1.13-1.23) | <0.001 |
| Total sleep duration, per hour increment | 0.98 (0.97-0.99) | <0.001 |

CI, confidence interval; NA, not applicable.

^a^ Hazard ratio was derived using Cox proportional hazard regression, which controlled for age, sex, ethnic background, education attainment, family income, employment status, alcohol consumption, physical activity, current smoking, history of chronic kidney disease, hypertension, diabetes, cancer, cardiovascular disease, and use of medications to lower blood pressure, glucose, as well as serum cholesterol. Total sleep duration was also adjusted.

**Supplemental Table 9.** Associations between daytime napping and incident atrial fibrillation, simultaneously controlling for all other sleep behaviors.

| **Exposures** | **Hazard ratio (95% CI) ^a^** | ***P* value** |
| --- | --- | --- |
| Daytime napping |  |  |
| Never/rarely | 1 (Reference) | NA |
| Sometimes | 1.07 (1.05-1.10) | <0.001 |
| Usually | 1.14 (1.09-1.19) | <0.001 |
| Daytime sleepiness |  |  |
| No | 1 (Reference) | NA |
| Yes | 1.12 (1.05-1.18) | <0.001 |
| Total sleep duration- per hour increment | 0.99 (0.98-1.00) | 0.083 |
| Early chronotype |  |  |
| No | 1 (Reference) | NA |
| Yes | 0.98 (0.96-1.00) | 0.088 |
| Insomnia symptoms |  |  |
| No | 1 (Reference) | NA |
| Yes | 1.10 (1.07-1.13) | <0.001 |
| Snoring |  |  |
| No | 1 (Reference) | NA |
| Yes | 1.01 (0.98-1.03) | 0.621 |

CI, confidence interval; NA, not applicable.

^a^ Hazard ratio was derived using Cox proportional hazard regression, which controlled for age, sex, ethnic background, education attainment, family income, employment status, alcohol consumption, physical activity, current smoking, history of chronic kidney disease, hypertension, diabetes, cancer, cardiovascular disease, and use of medications to lower blood pressure, glucose, as well as serum cholesterol. All other sleep behaviors were simultaneously adjusted, including daytime sleepiness, total sleep duration, chronotype (early chronotype or not), insomnia symptoms (whether usually encounters insomnia symptoms), and snoring.

**Supplemental Table 10.** Associations between daytime napping and incident atrial fibrillation, excluding events within two years since baseline.

| **Exposures** | **Events/total** | **Hazard ratio (95% CI) ^a^** | ***P* value** |
| --- | --- | --- | --- |
| Daytime napping | | | |
| Never/rarely | 13 722/267 001 | 1 (Reference) | NA |
| Sometimes | 13 533/180 269 | 1.08 (1.05-1.10) | <0.001 |
| Usually | 2545/24 466 | 1.15 (1.10-1.20) | <0.001 |
| Linear trend | 29 800/471 736 | 1.07 (1.05-1.09) | <0.001 |

CI, confidence interval; NA, not applicable.

^a^ Hazard ratio was derived using Cox proportional hazard regression, which controlled for age, sex, ethnic background, education attainment, family income, employment status, alcohol consumption, physical activity, current smoking, history of chronic kidney disease, hypertension, diabetes, cancer, cardiovascular disease, and use of medications to lower blood pressure, glucose, as well as serum cholesterol.

**Supplemental Table 11.** Associations between daytime napping and incident atrial fibrillation, accounting for competing risk from death using Fine-Gray sub-distribution hazard model.

| **Exposures** | **Events/competing events/total** | **Hazard ratio (95% CI) ^a^** | ***P* value** |
| --- | --- | --- | --- |
| Daytime napping | | | |
| Never/rarely | 14 837/14 881/269 038 | 1 (Reference) | NA |
| Sometimes | 14 752/14 958/182 531 | 1.08 (1.05-1.10) | <0.001 |
| Usually | 2835/3074/25 019 | 1.14 (1.10-1.19) | <0.001 |
| Linear trend | 32 424/32 913/476 588 | 1.07 (1.05-1.09) | <0.001 |

CI, confidence interval; NA, not applicable.

^a^ Hazard ratio was derived using Cox proportional hazard regression, which controlled for age, sex, ethnic background, education attainment, family income, employment status, alcohol consumption, physical activity, current smoking, history of chronic kidney disease, hypertension, diabetes, cancer, cardiovascular disease, and use of medications to lower blood pressure, glucose, as well as serum cholesterol.

**Supplemental Table 12.** Associations between daytime napping and incident atrial fibrillation, further controlling for body mass index.

| **Exposures** | **Events/total** | **Hazard ratio (95% CI) ^a^** | ***P* value** |
| --- | --- | --- | --- |
| Daytime napping | | | |
| Never/rarely | 14 770/268 231 | 1 (Reference) | NA |
| Sometimes | 14 658/181 742 | 1.04 (1.02-1.07) | <0.001 |
| Usually | 2799/24 803 | 1.11 (1.06-1.15) | <0.001 |
| Linear trend | 32 227/474 776 | 1.05 (1.03-1.07) | <0.001 |

CI, confidence interval; NA, not applicable.

^a^ Hazard ratio was derived using Cox proportional hazard regression, which controlled for age, sex, ethnic background, education attainment, family income, employment status, alcohol consumption, physical activity, current smoking, history of chronic kidney disease, hypertension, diabetes, cancer, cardiovascular disease, and use of medications to lower blood pressure, glucose, as well as serum cholesterol. Body mass index was also adjusted.

**Supplemental Table 13.** Associations between daytime napping and incident atrial fibrillation, further controlling for Fried frailty phenotype.

| **Exposures** | **Events/total** | **Hazard ratio (95% CI) ^a^** | ***P* value** |
| --- | --- | --- | --- |
| Daytime napping | | | |
| Never/rarely | 14 770/268 231 | 1 (Reference) | NA |
| Sometimes | 14 658/181 742 | 1.07 (1.04-1.09) | <0.001 |
| Usually | 2799/24 803 | 1.12 (1.07-1.16) | <0.001 |

CI, confidence interval; NA, not applicable.

^a^ Hazard ratio was derived using Cox proportional hazard regression, which controlled for age, sex, ethnic background, education attainment, family income, employment status, alcohol consumption, physical activity, current smoking, history of chronic kidney disease, hypertension, diabetes, cancer, cardiovascular disease, and use of medications to lower blood pressure, glucose, as well as serum cholesterol. Fried frailty phenotype was also adjusted.

**Supplemental Table 14.** Associations between daytime napping and incident atrial fibrillation, further controlling for night shift work schedule.

| **Exposures** | **Events/total** | **Hazard ratio (95% CI) ^a^** | ***P* value** |
| --- | --- | --- | --- |
| Daytime napping | | | |
| Never/rarely | 14 837/269 038 | 1 (Reference) | NA |
| Sometimes | 14 752/182 531 | 1.08 (1.05, 1.10) | <0.001 |
| Usually | 2835/25 019 | 1.17 (1.12, 1.21) | <0.001 |
| Linear trend | 32 424/476 588 | 1.08 (1.06, 1.10) | <0.001 |
| Night shift work | | | |
| Not involve shift work | 9707/227 034 | 1 (Reference) | NA |
| Never/rarely | 1049/23 120 | 1.08 (1.01, 1.15) | 0.017 |
| Sometimes | 591/13 566 | 1.09 (1.00, 1.19) | 0.043 |
| Usually | 499/10 512 | 1.15 (1.05, 1.26) | 0.003 |
| Not currently employed | \| 20 578/202 356 \| \| --- \| | 1.03 (1.00, 1.06) | 0.084 |

CI, confidence interval; NA, not applicable.

^a^ Hazard ratio was derived using Cox proportional hazard regression, which controlled for age, sex, ethnic background, education attainment, family income, employment status, alcohol consumption, physical activity, current smoking, history of chronic kidney disease, hypertension, diabetes, cancer, cardiovascular disease, and use of medications to lower blood pressure, glucose, as well as serum cholesterol. Night shift work schedule was also adjusted.

**Supplemental Table 15.** Non-response analysis comparing baseline characteristics of included and excluded participants in UK Biobank.

| **Characteristics ^a^** | **Excluded**  **N=25 781** | **Included**  **N=** **476 588** | ***P* ^b^** |
| --- | --- | --- | --- |
| Age, mean (SD), y | 57.9 (8.2) | 56.5 (8.1) | <0.001 |
| Men | 13 026 (50.5) | 216 042 (45.3) | <0.001 |
| White ethnicity | 22 503 (87.3) | 449 501 (94.3) | <0.001 |
| Education attainment | | | |
| Missing | 4797 (18.6) | 5335 (1.1) | <0.001 |
| Lower education | 4768 (18.5) | 80 489 (16.9) |  |
| Secondary education | 6887 (26.7) | 166 579 (35.0) |  |
| Higher education | 9329 (36.2) | 224 185 (47.0) |  |
| Annual household income, £ | | | |
| Missing | 8215 (31.9) | 68 923 (14.5) | <0.001 |
| <18 000 | 5364 (20.8) | 91 812 (19.3) |  |
| 18 000-30 999 | 4681 (18.2) | 103 462 (21.7) |  |
| 31 000-51 999 | 4044 (15.7) | 106 702 (22.4) |  |
| 52 000-100 000 | 2721 (10.6) | 83 522 (17.5) |  |
| >100 000 | 756 (2.9) | 22 167 (4.7) |  |
| Occupation | | | |
| Missing | 1078 (4.2) | 1775 (0.4) | <0.001 |
| Unemployed | 2680 (10.4) | 36 656 (7.7) |  |
| Employed | 22 023 (85.4) | 438 157 (91.9) |  |
| Chronic kidney disease | 1304 (5.1) | 12 676 (2.7) | <0.001 |
| Hypertension | 15 788 (61.2) | 264 639 (55.5) | <0.001 |
| Diabetes | 2573 (10.0) | 29 069 (6.1) | <0.001 |
| Cardiovascular disease | 9654 (37.4) | 30 698 (6.4) | <0.001 |
| Cancer | 3373 (13.1) | 49 967 (10.5) | <0.001 |
| Medication for blood pressure | 7133 (27.7) | 96 850 (20.3) | <0.001 |
| Medication for blood glucose | 1564 (6.1) | 17 617 (3.7) | <0.001 |
| Medication for serum cholesterol | 7182 (27.9) | 83 144 (17.4) | <0.001 |

^a^ Data represented characteristics as mean (SD) or n (%).

^b^ P value reported for differences between groups using t test or chi-squared test.


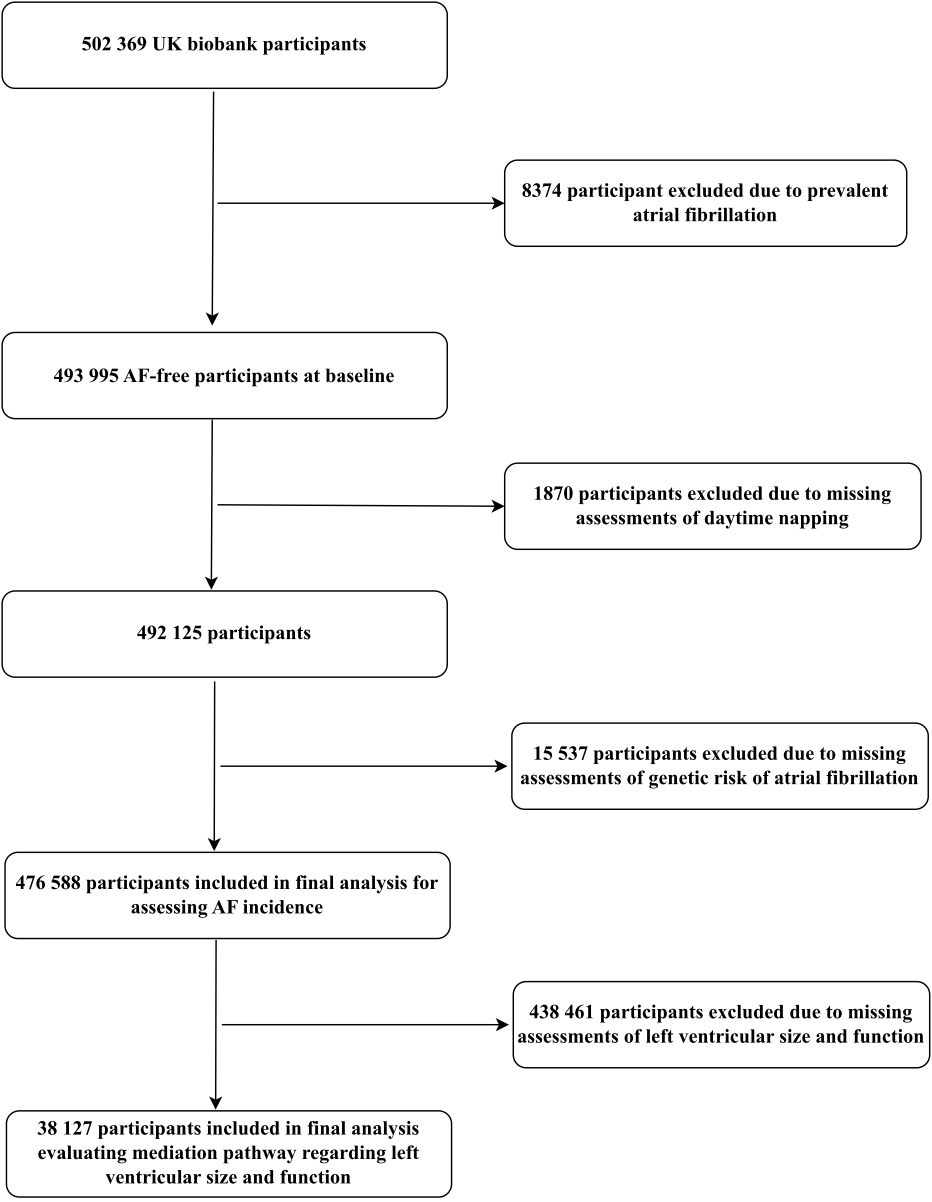


**Supplemental Figure 1.** Participants selection diagram.


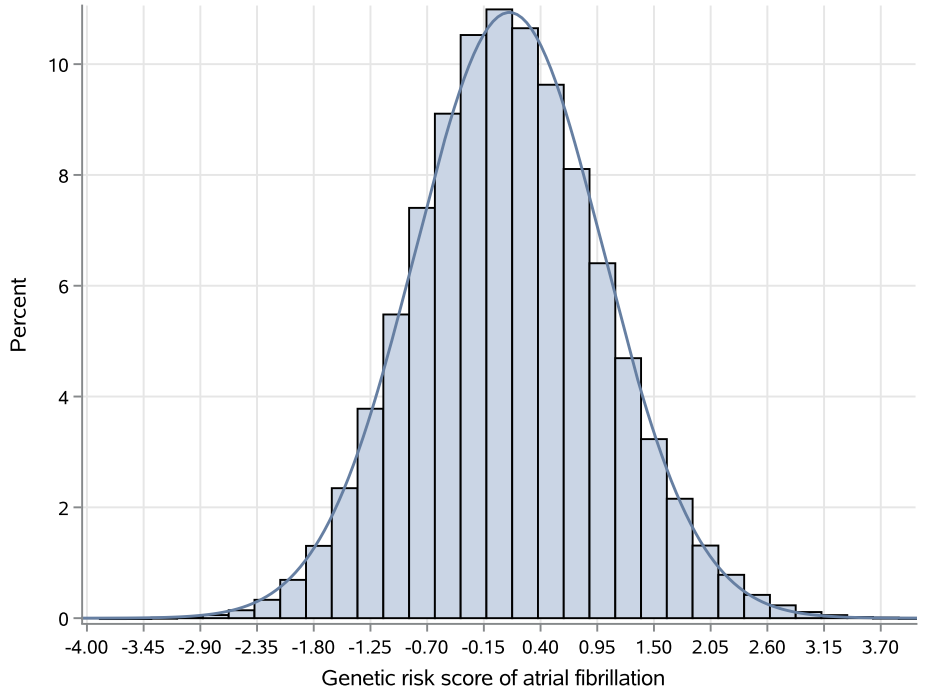


**Supplemental Figure 2.** Distribution of calculated genetic risk score of atrial fibrillation.


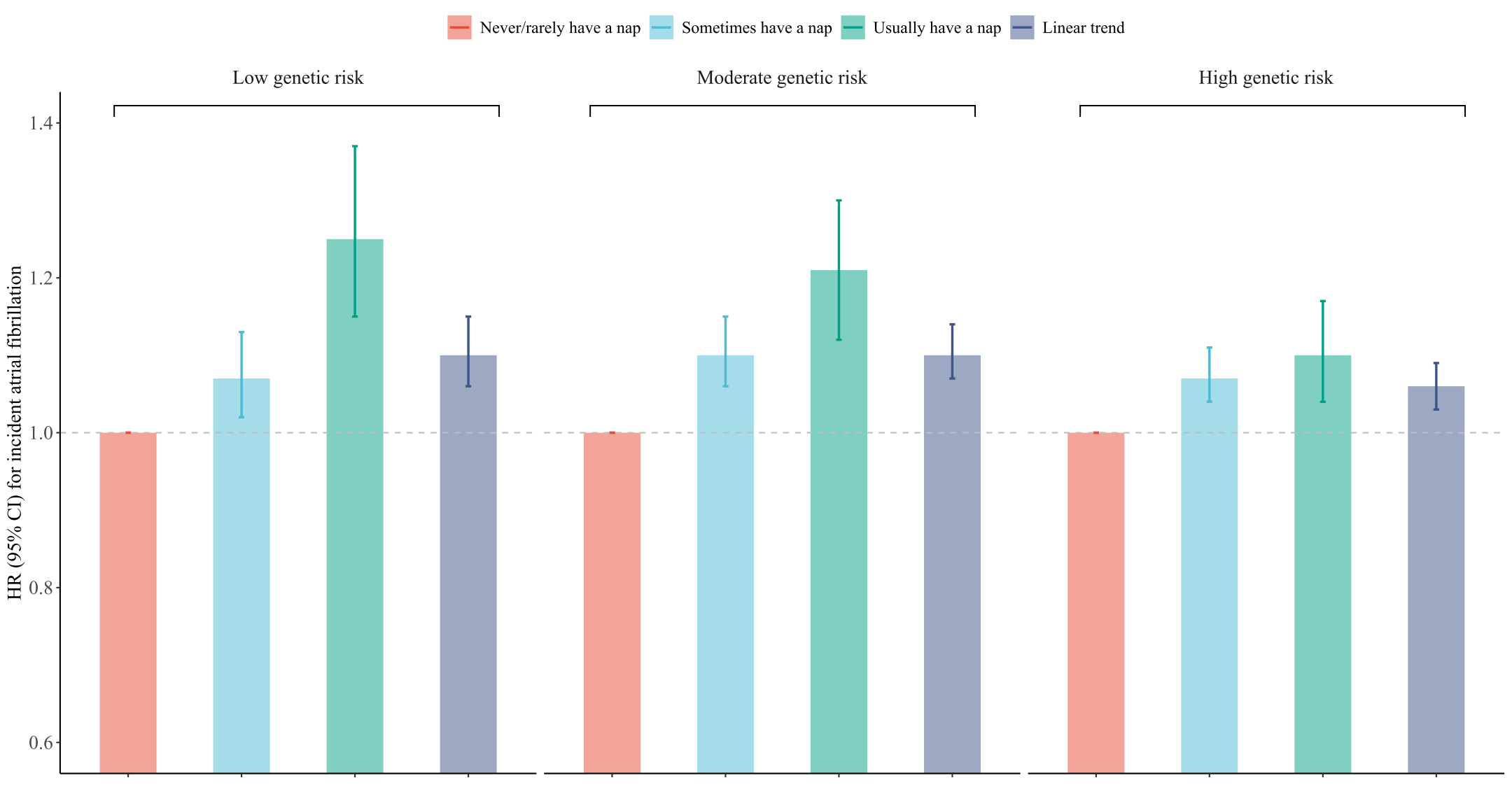


**Supplemental Figure 3.** Associations between daytime napping frequency and incident atrial fibrillation according to stratification of genetic risk.

HR, hazard ratio; CI, confidence interval.

Hazard ratios and 95% confidence intervals were obtained using Cox proportional hazard regression, which controlled for age, sex, ethnic background, education attainment, family income, employment status, alcohol consumption, physical activity, current smoking, history of chronic kidney disease, hypertension, diabetes, cancer, cardiovascular disease (other than atrial fibrillation), and use of medications to lower blood pressure, glucose, as well as serum cholesterol.


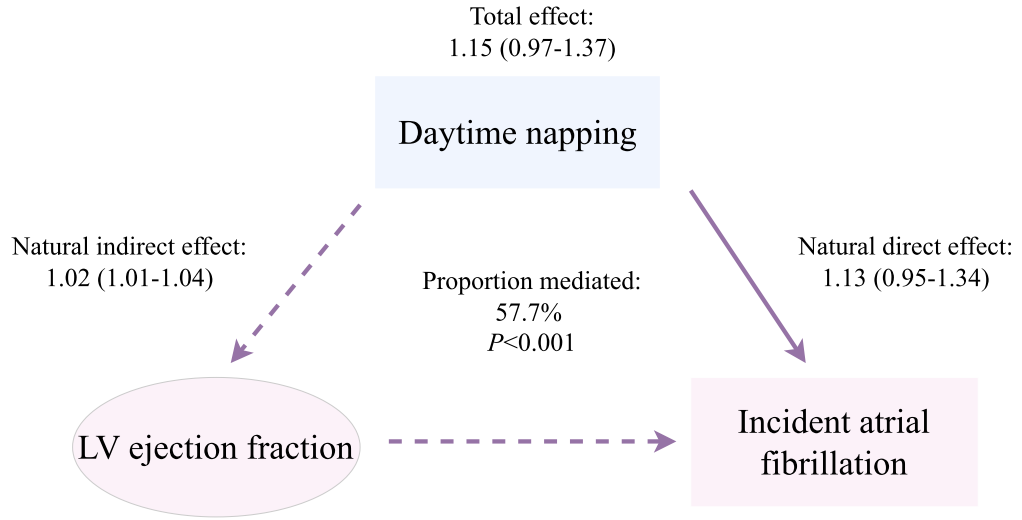


**Supplemental Figure 4.** Causal mediation analysis evaluating the mediation pathway of left ventricular ejection fraction in linking daytime napping with incident atrial fibrillation.

LV, left ventricular.

Causal mediation analysis based on the regression approach was conducted, with effect estimates expressed as odds ratios. Adjusted covariates included age, sex, ethnic background, education attainment, alcohol consumption, physical activity, current smoking, history of chronic kidney disease, hypertension, diabetes, cancer, cardiovascular disease (other than atrial fibrillation), and use of medication to lower blood pressure.


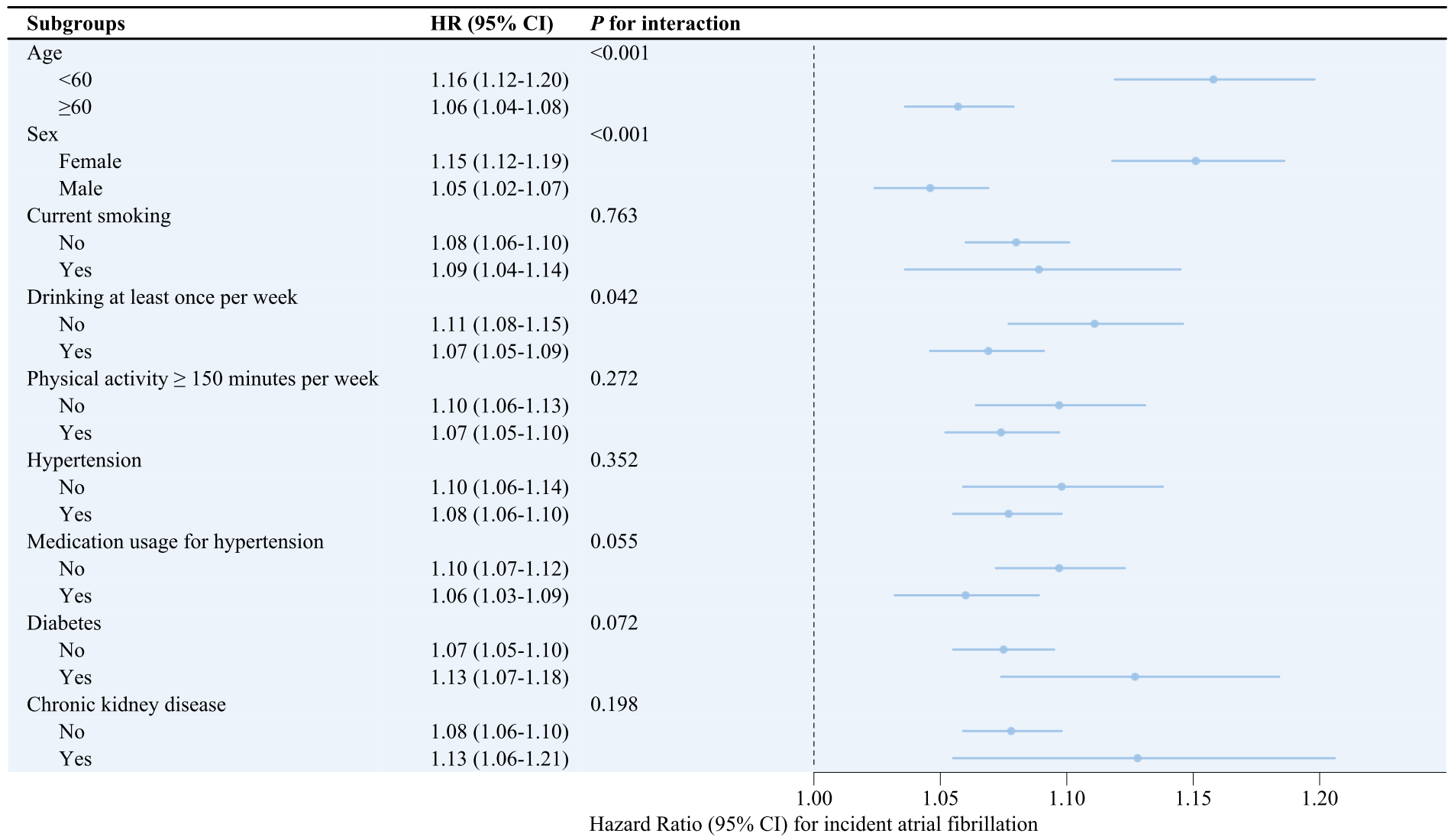


**Supplemental Figure 5.** Subgroup analysis to examine potential interactions between daytime napping and selected baseline covariates.

HR, hazard ratio; CI, confidence interval.

Hazard ratio was derived using Cox proportional hazard regression, which controlled for age, sex, ethnic background, education attainment, family income, employment status, alcohol consumption, physical activity, current smoking, history of chronic kidney disease, hypertension, diabetes, cancer, cardiovascular disease (other than atrial fibrillation), and use of medications to lower blood pressure, glucose, as well as serum cholesterol. P for interaction was calculated by adding a multiplicative interaction term between daytime napping frequency (as a linear term) and potential modifier (selected baseline covariates) in the Cox model.


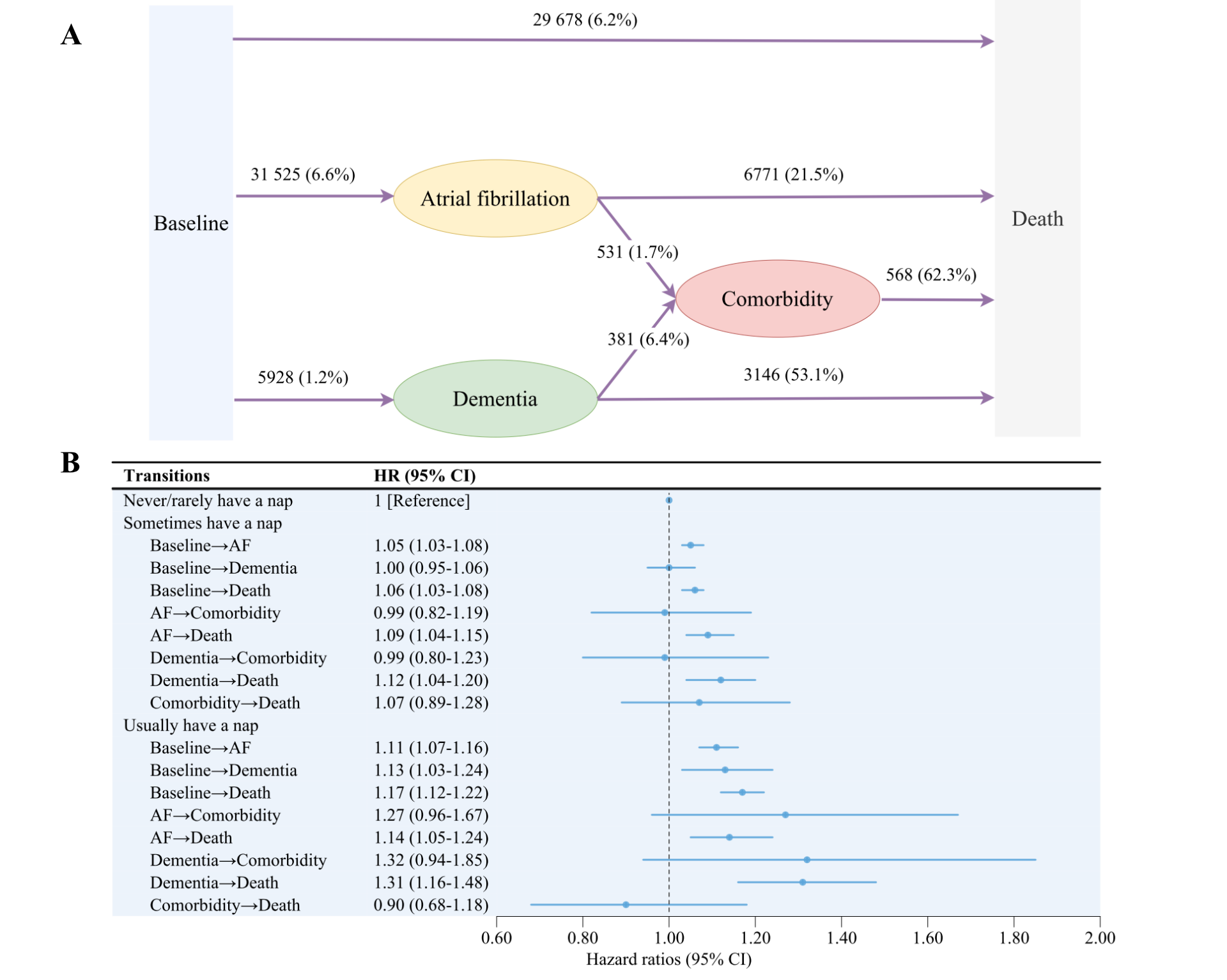


**Supplemental Figure 6.** Associations between napping and disease transition hazards of atrial fibrillation and dementia, excluding participants with dementia diagnosis within two years of incident atrial fibrillation.

AF, atrial fibrillation; HR, hazard ratio; CI, confidence interval.

(A) Observed transition patterns (expressed as number and percentages) of atrial fibrillation and dementia; (B) Hazard ratios of associations between daytime napping with different transition patterns of atrial fibrillation and dementia.


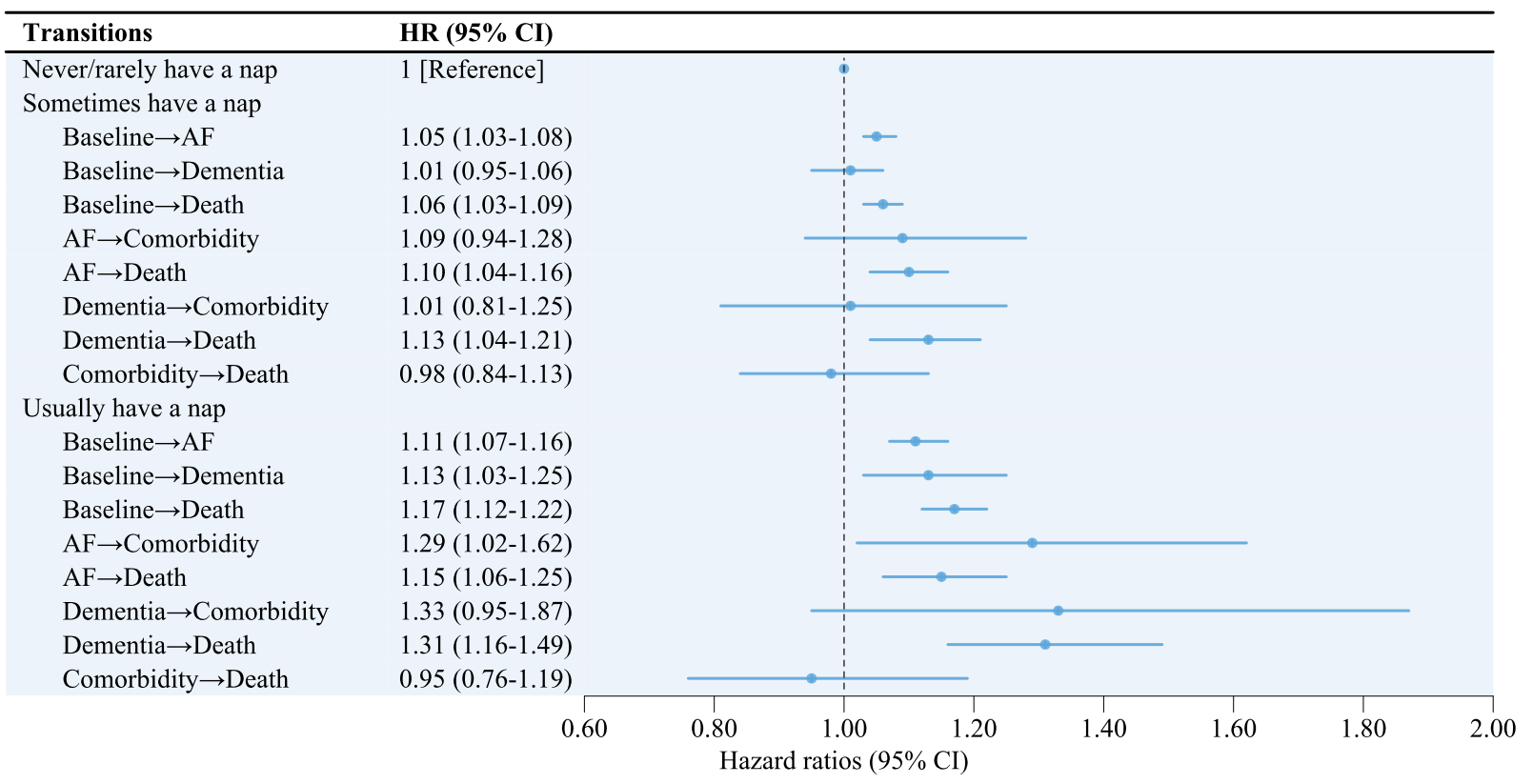


**Supplemental Figure 7.** Associations between napping and disease transition hazards of atrial fibrillation and dementia, further controlling for snoring.

AF, atrial fibrillation; HR, hazard ratio; CI, confidence interval.

Hazard ratios of associations between daytime napping with different transition patterns of atrial fibrillation and dementia.
